# Optimal Allocation of Observations in Stepped‐Wedge and Other Cluster Studies With Correlated Cluster‐Period Effects

**DOI:** 10.1002/sim.70100

**Published:** 2025-06-23

**Authors:** Alan J. Girling, Samuel I. Watson

**Affiliations:** ^1^ Institute of Applied Health Research University of Birmingham Birmingham UK

**Keywords:** cluster studies, optimal design, staircase designs, stepped wedge

## Abstract

Stepped‐wedge studies usually entail regular sampling of clusters over time. Yet the precision of the treatment effect estimator can sometimes be improved if the regular sampling scheme is replaced by one with preferential allocation of observations to particular time‐epochs within each cluster. We present some exact results for optimizing the allocation for a general experimental layout under a mixed effects model with a time‐varying cluster‐autocorrelation structure, together with an algorithm for generating optimal allocations. An index of cluster variation is introduced, an increasing function of both the intra‐class correlation and the total sample size, which encapsulates the influence of cluster‐level variation on the optimal allocation. For any specified layout there is a sampling scheme (the ‘best natural allocation’) that solves the optimization problem for all values of this index up to a threshold value which depends only on the cluster autocorrelations. Under such a scheme the treatment effect estimator is equal to a simple difference between the means of the treated and control observations. Best natural allocations stand alongside conventional parallel and cross‐over designs in giving equal weight to observations from all participants, even under stepped‐wedge layouts with irreversible interventions. When applied to a recent study of primary care training programmes in low‐ and middle‐ income countries (The REaCH study), the results lead to substantial reductions in total sample size, without loss of precision. For stepped‐wedge layouts with block‐exchangeable or time‐decaying cluster autocorrelations, we present explicit conditions for the optimality of staircase‐type sampling schemes, which can arise as best natural allocations in such cases.

## 1 Introduction

The stepped‐wedge design has received some attention over the last few years for trials of cluster‐level interventions where the intervention is irreversible—that is, it cannot readily be withdrawn once it has been introduced into a cluster—and where it is desirable that all clusters receive the intervention during the course of the trial [1, 2]. For example, it offers an opportunity for evaluation during the roll‐out of a service‐delivery intervention which would otherwise lack a controlled assessment [3]. Nevertheless, it has become apparent that the conventional ‘complete’ stepped‐wedge layout—where each cluster returns the same number of observations in each of a sequence of time‐epochs defined by the treatment switch points—may not be the most efficient version of the design [4]. Approaches to optimize the design are reviewed by Watson, Hemming, and Girling [4]. Much of this work focusses on complete designs, seeking either to optimize the switch‐times at which the treatment is introduced [5, 6, 7], or to optimize the distribution of clusters among a set of sequences with pre‐specified treatment regimes [5, 8, 9]. Further, it has been noted that there can be a wide variation in the information content between the cells of a complete design [10], as measured by their contribution to the treatment‐effect estimator, so that the design can be improved by concentrating the sampling effort on the most influential cells [11]. More generally, it suggests that a redistribution of observations between cells could be advantageous.

Our work is motivated by this insight but is not restricted to conventional stepped‐wedge layouts. The results in Section 4 apply to experimental layouts whose rows consist of specified cluster‐sequences, where a cluster‐sequence is defined as a temporal series of experimental conditions in discrete time. Examples could include parallel cluster designs (where clusters are assigned either to a Control or a Treated sequence), cross‐over designs (where Treated and Control periods alternate within each sequence) and variants of stepped‐wedge designs with washout periods and/or repeated treatment patterns.

We address explicitly the design of a study consisting of a single replicate of a basic layout—that is, one in which each row of the layout accounts for just one cluster. Note that layouts with duplicated rows are not excluded, in which case more than one cluster will share the same treatment‐sequence. Within the basic layout, we seek to allocate a fixed number of experimental units (observations) among the cluster‐periods so as to maximize the precision of the treatment‐effect estimator. Our approach is directed toward finding the optimal proportional allocation of units, in percentage terms, among the cells of the layout. The solution may not be fully realizable in practice where only whole numbers of units can be allocated, but will often suggest approximate optimal designs. The results are directly transferable to replicated designs where equal numbers of clusters are allocated to each row of the layout. In such cases, the precision of the study will be appropriately scaled (by the number of replicates), but the relative performance of different choices for the basic allocation is unaffected. A choice that is optimal for a single replicate must be optimal in the replicated design. To this extent, the results are relevant to the common situation where the clusters outnumber the rows of the layout.

A linear mixed model is assumed throughout, with a general cluster correlation structure and a fixed effect for each time‐epoch. The optimal allocation depends on two groups of parameters: (I) the total number of observations in the basic layout, N, together with the Intra‐cluster correlation (ICC), ρ; and (II) the cluster autocorrelations (CACs) that govern the cluster‐period components in the mixed‐effects model. The parameters N and ρ in group (I) are combined in an index of cluster variation (ICV), S, which entirely encapsulates their influence on the optimal allocation. We show that the CACs in (II) determine an allocation—the “Best Natural Allocation”—for which the treatment effect is estimated as a simple difference of means, and which is optimal for all values of the ICV up to, and including, a threshold‐value. Above the threshold the optimal allocation is sensitive to the precise values of the ICV. Nevertheless, the threshold is often large enough to suggest that the best natural allocation will be optimal or close to optimal in many practical studies. Under a best natural allocation, each observation contributes the same amount of information to the treatment effect estimator. This feature is shared with many standard trial designs (e.g., balanced parallel and cross‐over cluster trials, and balanced individually randomized trials). It holds even under a stepped‐wedge treatment layout where observations in different cells would otherwise contribute disproportionately to the effect estimate. To this extent, best natural allocations under irreversible treatment regimes stand alongside conventional trial designs in giving equal weight to all trial participants.

For stepped‐wedge layouts, the solutions found are not ‘complete’ designs [12]—typically they include some empty cells with no observations, with unequally‐sized samples taken in the other cells. For example, the best natural allocation under a stepped‐wedge layout has a ‘staircase’ structure when the cluster autocorrelations decay slowly over time. (The details are spelt out in Section 6.) A direct comparison can be drawn with the computational algorithms described by Watson and others [4, 13] which also lead to staircase solutions in some cases. In related work, Hooper et al. [14] seek to optimize both the treatment switch times within each cluster and the times at which observations are made, under a linear mixed model with a polynomial time‐effect. Here we have retained a discrete‐time framework which guarantees solutions with contemporaneous controls and gives access to some new (exact) results on the structure of the optimal designs.

The paper is organized as follows. The general analytical approach, including the statistical model and a numerical algorithm, is described in Sections 2 and 3. Section 4 contains the main results on the optimal properties of best natural allocations. The remainder of the paper is explicitly concerned with study design over a stepped‐wedge layout, including some alternative optimized designs for the ReACH study in Section 5. Staircase solutions are derived in Section 6, together with exact conditions for their optimality. Detailed mathematical arguments are reserved for the Appendices. The paper concludes with some further discussion.

## 2 Notation and Model

**FIGURE 1:**
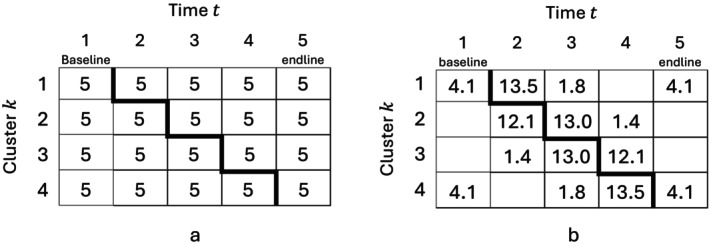
Optimizing a stepped‐wedge design. (a) shows a standard stepped wedge design for 4 clusters with 5% of the observations in each cell. At baseline (t=1) all clusters are in the control condition. Treated cells lie north and east of the bold border lines so that all clusters are in the treated condition at endline (t=5). Using the algorithm described in the text, the observations in (b) have been re‐allocated among the cells to optimize the design under a BEX model with, $\Gamma_{\mathrm{st}}=0.7+0.3\delta_{\mathrm{st}}$ and $N\rho /\left(1-\rho \right)=26.\dot{6}$ (i.e., S=0.87). Empty cells—that is those with ${\tilde{\pi }}_{\mathrm{kt}}<10^{-5}$– are left blank. The efficiency of the standard design relative to the optimized design is 73%.

We focus explicitly on the design of a study in which there are K cluster‐sequences with one cluster per sequence. The layout ℒ is defined as the subset of a K×T lattice on which the cluster‐sequences are specified. For (k,t)∈ℒ, let $Z_{\mathrm{kt}}$ denote the (fixed) treatment‐status of the *k*th cluster‐sequence (k∈{1,…,K}) at time t∈{1,…,T} with $Z_{\mathrm{kt}}=1$ if the treatment‐condition is present, and $Z_{\mathrm{kt}}=0$ if the control‐condition is present. One further stipulation is necessary: for at least one time‐point t there must exist cluster‐sequences k and $k^{\prime }$ for which $Z_{\mathrm{kt}}=1$ and $Z_{k^{\prime }t}=0$, so that a controlled treatment comparison is possible. The motivating examples occur where the cluster‐sequences generate a standard stepped‐wedge layout, in which case ℒ is the complete set of lattice points with T=K+1 and $Z_{\mathrm{kt}}=I_{\left\{t>k\right\}}$ for all (k,t), and the treatment assignments represent an irreversible intervention that is introduced into the *k*th cluster‐sequence at time k+1 (as in Figure 1a).

For included cluster‐periods (k,t)∈ℒ, we specify a mixed effects model for the *i*th observation at time t from the *k*th cluster as:

$$y_{\mathrm{kti}}=\beta_{t}+\theta Z_{\mathrm{kt}}+\gamma_{\mathrm{kt}}+\varepsilon_{\mathrm{kti}};k=1,\ldots ,K,t=1,\ldots ,T,i=1,\ldots ,n_{\mathrm{kt}},$$

where $n_{\mathrm{kt}}$ is the number of experimental observations in the *k*th cluster at time t, typically from different subjects in a cross‐sectional study, and $\sum_{\left(k,t\right)\in ℒ}n_{\mathrm{kt}}=N$, the total number of observations in the basic layout. The parameter $\beta_{t}$ represents an unknown fixed time effect that applies in all clusters at time t, and θ is the treatment effect parameter. The random cluster effects, $\gamma_{\mathrm{kt}}$, are independent between different clusters, with covariance structure within clusters given by $\mathrm{cov}\left(\gamma_{\mathrm{ks}},\gamma_{\mathrm{kt}}\right)={\rho \sigma }^{2}\Gamma_{\mathrm{st}},\left(s,t=1,\ldots ,T\right)$ where $\sigma^{2}=\mathrm{var}y_{\mathrm{kti}}=\mathrm{var}\gamma_{\mathrm{kt}}+\mathrm{var}\varepsilon_{\mathrm{kti}}$, ρ is the within‐period ICC and Γ is a Toeplitz correlation matrix (i.e., $\Gamma_{\mathrm{st}}$ depends only on |s−t|) of CACs that describe the cluster‐level effects. The variance of the residual error term is $\mathrm{var}\left(\varepsilon_{\mathrm{kti}}\right)=\left(1-\rho \right)\sigma^{2}$.

Where a specific form is needed for Γ, we use a two‐parameter family (“the Γ‐family”) of the form.

(1) $$\Gamma_{\mathrm{st}}=\alpha r^{\left|s-t\right|}+\left(1-\alpha \right)\delta_{\mathrm{st}},\text{for}\;s,t=1,\ldots T,\text{and}\;0\leq \alpha ,r\leq 1,$$

where $\delta_{\mathrm{st}}=1$ or 0 according as s=t or s≠t. This generates the correlation structure described by Kasza et al. [15]. Such models allow for decay in the correlation between observations in different time‐periods, s and t. The case α=1,r=1 corresponds to the Exchangeable (EXC) model [3] in which the correlation is the same between any two observations in the same cluster. Otherwise, r=1,0<α<1 gives the block‐exchangeable (BEX) model with exchangeable time‐periods within clusters [5, 12]; and α=1 with 0<r<1 gives a discrete‐time decay model (DTD) [15, 16]. If α=0 or r=0, then Γ=I, the unit matrix, and there is no correlation between different cluster‐periods.

We define an Index of Cluster‐level Variation (ICV) by

(2) $$S=\frac{M\rho }{1+\left(M-1\right)\rho },$$

where M=N/K is the average cluster‐size. The ICV captures a combination of the ICC and cluster size which has previously been found to play an important role for optimizing the treatment switch points under the EXC‐model with identical cell sizes [5]. In that context the ICV is equivalent to the cluster‐mean correlation.

Our results are directed towards optimizing the sample sizes within each cluster‐period—that is the *n*_*kt*_s—subject to a specified total number of observations, N, assuming that there will be exactly one cluster per sequence. In practical studies it may happen that more than one cluster is assigned to each cluster‐sequence. This may be unavoidable—for example, where the length of the study is too short to permit a full stepped‐wedge layout with the desired number of clusters. In other cases, a degree of replication can serve as a precaution against possible sampling difficulties in individual clusters which could lead to missing cluster‐sequences at the analysis stage. For example, if exactly G×K clusters are available (for some G), with G×N observations in total, the clusters can be assigned to the rows of the experimental layout in equal groups of size G, thus replicating the optimal design with K clusters. Under such an assignment the variance of the treatment effect estimator would be reduced by the factor $G^{-1}$. An extension of the optimal design problem would allow for variation in both the cell contents and the numbers of clusters assigned to each cluster‐sequence, but this is not considered here. The current work applies only to studies with one or more complete replicates of a single layout with K sequences and N observations per replicate.

## 3 Methodology: Finding Optimal Designs

We suppose that the total number of observations, N, is fixed. The design of the study is identified with the K×T array n, defined on ℒ, and an optimal design $\tilde{n}$ is a design for which the variance of the Best Linear Unbiased Estimator (BLUE) of θ is a minimum. We define the “allocation” π corresponding to the design n by $\pi_{\mathrm{kt}}=n_{\mathrm{kt}}/N\;\text{for}\;\left(k,t\right)\in ℒ$, where $\pi_{\mathrm{kt}}$ is the proportion of the total sample allocated to cell (k,t). We focus on the optimal allocation, regarding the $\pi_{\mathrm{kt}}$s as a collection of continuous quantities so that the results will sometimes apply only approximately to practical design problems where only integer $n_{\mathrm{kt}}$s are allowed.

The BLUE of θ takes the form

$$\hat{\theta }=\sum_{\left(k,t\right)\in \mathrm{supp}\left(\pi \right)}a_{\mathrm{kt}}{\bar{y}}_{\mathrm{kt}}$$

where the sum is taken over the support of π—the set of points in ℒ for which $\pi_{\mathrm{kt}}\neq 0$—and ${\bar{y}}_{\mathrm{kt}}=n_{\mathrm{kt}}^{-1}\sum_{i=1}^{n_{\mathrm{kt}}}y_{\mathrm{kti}}$, the mean of the observations in cell (k,t). For empty cells—that is those with $\pi_{\mathrm{kt}}=0$—we choose to set the coefficient $a_{\mathrm{kt}}=0$. Also, to eliminate the time‐parameters $\left(\beta_{t}\right)$ and ensure that $\hat{\theta }$ is unbiased for θ), we must have.

(3) $$a\in 𝒜=\left\{a;\sum_{\underset{\left(k,t\right)\in ℒ}{k=1}}^{K}a_{\mathrm{kt}}=0\;\text{for}\;\mathrm{all}\;t=1,\ldots ,T,\sum_{\left(k,t\right)\in ℒ}a_{\mathrm{kt}}Z_{\mathrm{kt}}=1\right\}.$$

Together these conditions mean that $a\in {𝒜}_{\pi }$ where

$${𝒜}_{\pi }=𝒜\cap \left\{a;\mathrm{supp}\left(a\right)\subset \mathrm{supp}\left(\pi \right)\right\}.$$

The BLUE coefficients $\left(a_{\mathrm{kt}}\right)$ are determined by minimizing the variance

(4) $$\begin{array}{ll} V\left(a,n\right) & =V\left(a,N\pi \right)=\mathrm{var}\left(\sum_{ℒ}a_{\mathrm{kt}}{\bar{y}}_{\mathrm{kt}}\right)\\ & \;=\sigma^{2}\left[\rho \sum_{k=1}^{K}\sum_{\left(k,s\right)\in ℒ}\sum_{\left(k,t\right)\in ℒ}a_{\mathrm{ks}}\Gamma_{\mathrm{st}}a_{\mathrm{kt}}+\frac{1-\rho }{N}\sum_{\left(k,t\right)\in \mathrm{supp}\left(\pi \right)}\frac{a_{\mathrm{kt}}^{2}}{\pi_{\mathrm{kt}}}\right] \end{array}$$

over $a\in {𝒜}_{\pi }$, for a fixed n=Nπ. The BLUE of θ is equivalent to the estimator obtained by the method of generalized least squares [17].

For fixed N, an optimal allocation $\tilde{\pi }$—with its associated array of BLUE coefficients $\tilde{a}$—is obtained by minimizing V(a,Nπ) in (4) over both a∈𝒜 and π, subject to the constraints

$$\pi_{\mathrm{kt}}\geq 0\;\text{for}\;\mathrm{all}\;\left(k,t\right)\in ℒ;\sum_{\left(k,t\right)\in ℒ}\pi_{\mathrm{kt}}=1;\mathrm{supp}\left(a\right)\subset \mathrm{supp}\left(\pi \right).$$

For any given a, the minimisation over π entails only the second term in square brackets in (4) and is accomplished by setting.

(5) $$\pi_{\mathrm{kt}}=\frac{\left|a_{\mathrm{kt}}\right|}{\sum_{\left(j,s\right)\in ℒ}\left|a_{\mathrm{js}}\right|}\;\text{for}\;\left(k,t\right)\in ℒ,$$

as shown in Appendix A.

The expression for $\pi_{\mathrm{kt}}$ in (5) can be substituted into (4) to obtain:

(6) $$\Psi \left(a\right)=\sigma^{-2}\min_{\pi }V\left(a,N\pi \right)=\rho \sum_{k=1}^{K}\sum_{\left(k,s\right)\in ℒ}^{T}\sum_{\left(k,t\right)\in ℒ}^{T}a_{\mathrm{ks}}\Gamma_{\mathrm{st}}a_{\mathrm{kt}}+\frac{1-\rho }{N}{\left(\sum_{\left(k,t\right)\in ℒ}\left|a_{\mathrm{kt}}\right|\right)}^{2}.$$

In principle, the coefficients $\left({\tilde{a}}_{\mathrm{kt}}\right)$ of the BLUE of θ under the optimal allocation can be got by minimizing the objective function, Ψ(a), over a∈𝒜. The optimal allocation $\left(\tilde{\pi }\right)$ follows by setting $a=\tilde{a}$ in (5) above, with a corresponding optimal design whose components are ${\tilde{n}}_{\mathrm{kt}}=N{\tilde{\pi }}_{\mathrm{kt}}$. From (5) it is evident that the (absolute) weight given to ${\bar{y}}_{\mathrm{kt}}$ in the estimator of θ is proportional to the size of the sample in cell (k,t). This means that, under an optimal allocation, each observation will have the same influence on the treatment effect estimator.

In realizing the optimal design—and depending on the value of N—it is likely that at least some of the ${\tilde{n}}_{\mathrm{kt}}$s will take non‐integer values. If desired, a rounding procedure can be applied to achieve a realizable approximation to the optimum, as in Pukelsheim and Rieder [18]. In any case, the ${\tilde{n}}_{\mathrm{kt}}$s can be treated as targets for recruitment in each cluster‐period even if they cannot be realized exactly in a given instance.

It is clear that $\tilde{\pi }$ depends on N and ρ only through the ratio of the coefficients of the two terms in (6), namely Nρ/(1−ρ), which is equal to KS/(1−S), from the definition of the ICV in (2). The same approach can be used to optimize the design over any subset of the layout, ℒ—that is when some of the cells are unavailable for sampling. In such cases the values of $a_{\mathrm{kt}}$ for prohibited cells are set to zero throughout the minimisation process.

The search for the optimal BLUE coefficients by minimizing Ψ(a) is a convex optimisation problem, for which any local minimum automatically corresponds to a global optimum. Furthermore, if the autocorrelation matrix Γ is of full rank (i.e., invertible) and ρ>0 it follows that the first summation in (6), and hence Ψ(a) itself, is a strictly convex function of a; then the optimal allocation is unique. In fact, Γis invertible for all values of α and r in the Γ‐family defined above (1), with the single exception of the EXC model, where α=1and r=1, and for which uniqueness is not guaranteed.

For fixed N, the performance of any allocation, π, can be assessed by comparing the precision attained by the BLUE of θ under π with that achievable under the optimal allocation, $\tilde{\pi }$. The efficiency of π is defined by

$$\mathrm{Eff}\left(\pi \right)=\frac{\mathrm{var}\left(\hat{\theta }|\text{optimal allocation},\tilde{\pi }\right)}{\mathrm{var}\left(\hat{\theta }|\text{allocation}\;\pi \right)}\leq 1.$$

It follows from (4) and (6) that Eff(π) depends on N and ρ only through the ratio Nρ/(1−ρ), which is itself a function ICV, S.

### 3.1 An Algorithm

Where an analytical solution is not available, optimal allocations can be found numerically.

For r=0,1,2,… we use 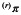 to denote the allocation at iteration r, and 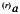 the corresponding array of BLUE coefficients.

For fixed N, the proposed algorithm proceeds from an initial candidate allocation πkt(0);(k,t)∈ℒ,∑ℒπkt=1, by applying the following steps for r=0,1,2,…

Step 1. Compute the BLUE coefficients (akt(r);(k,t)∈ℒ) for allocation πkt(r). This is most easily done by finding the coefficients of the generalized least squares estimator of θ, which is equivalent to the BLUE.

Step 2. Update the allocation by setting

πkt(r+1)=|akt(r)|∑(l,s)∈ℒ|als(r)|for(k,t)∈ℒ

Steps 1 and 2 are repeated until convergence is achieved. The algorithm is described more fully in Appendix I. The results in this paper were obtained from coding in Mata, within the Stata package v16 (StataCorp, College Station, TX USA), which is attached in a Supplemental file. A general version of the algorithm is also available as part of the R package glmmrOptim. The algorithm is integrated into an online cluster trial design application at https://www.clustertrial.app [19].

Step 1 minimizes V(a,Nπ(r)) over a, for fixed π(r); and step 2 minimizes V(a(r),Nπ) over n, for fixed a(r). The function V(a,Nπ) decreases (or at least does not increase) at each step of the procedure, which is therefore guaranteed to converge. Nevertheless, it may sometimes fail to reach an optimal allocation. For example, a cell (k,t) that is empty at step r—that is πkt(r)=0—must also be empty at all subsequent steps—that is πkt(s)=0 for all s>r—and cannot feature in any limiting design. Despite this limitation, the algorithm seems to be an effective way to find optimal allocations where analytical optimisation is not feasible, provided that the initial candidate design contains non‐zero entries in every available cell. An example for a stepped‐wedge layout is given in Figure 1.

## 4 Optimal Natural Allocations

Many standard designs are balanced in the sense that the same number of observations is assigned to both the treatment and control conditions. For such designs, a naïve estimate of the treatment effect might be given as the difference between the means of the treated and control observations. In our notation this would be

$${\hat{\theta }}_{\text{naive}}=2\left(\sum_{ℒ}\pi_{\mathrm{kt}}Z_{\mathrm{kt}}{\bar{y}}_{\mathrm{kt}}-\sum_{ℒ}\pi_{\mathrm{kt}}\left(1-Z_{\mathrm{kt}}\right){\bar{y}}_{\mathrm{kt}}\right).$$

In general, we say that a balanced design follows a ‘natural’ allocation if the naïve mean difference estimator is actually the BLUE for θ under the mixed effects model.

More formally, an allocation π is a Natural Allocation (NA) on ℒ if the corresponding BLUE coefficients (i.e., that minimize (4)) are given by

$$a_{\mathrm{kt}}^{\left(\pi \right)}=2\left(2Z_{\mathrm{kt}}-1\right)\pi_{\mathrm{kt}},\text{for}\;\left(k,t\right)\in ℒ.$$

Under any NA the treatment effect estimator takes the mean–difference form given above.

Since $a^{\left(\pi \right)}\in 𝒜$, necessarily, the coefficients must satisfy $\sum_{\left\{k;\left(k,t\right)\in ℒ\right\}}a_{\mathrm{kt}}^{\left(\pi \right)}=0$ for t=1,…,T, from which it follows that $\sum_{k}Z_{\mathrm{kt}}\pi_{\mathrm{kt}}=\sum_{k}\left(1-Z_{\mathrm{kt}}\right)\pi_{\mathrm{kt}}$. In other words, where the allocation is a natural one, the design is balanced over treatment assignment at every time‐point, not just overall, as in Figure 1b. One consequence is that any NA must be confined to time‐epochs at which both treated and control cluster‐periods are present. For example, the baseline (T=1) and endline (T=K+1) epochs of a stepped‐wedge layout cannot feature in any such allocation. NAs have an obvious intuitive appeal in that all observations contribute equally to a readily computable—and interpretable—treatment effect.

The following properties are proved in Appendix B.

### 4.1 Properties of Natural Allocations

Suppose that π is an allocation over a layout ℒ.

- Whether π is a NA or not depends on the cluster autocorrelation matrix, Γ, but is independent of the total sample size N and the ICC, ρ. In particular, it is independent of the ICV, S.
- If π is a NA, it is the optimal allocation over its own support for all values of N and ρ. That is π is optimal over the sub‐layout $\left\{\left(k,t\right)\in ℒ;\pi_{\mathrm{kt}}>0\right\}=\mathrm{supp}\left(\pi \right)$.
- If π is a NA the treatment effect estimator has variance
$$\mathrm{var}\;\hat{\theta }=\sigma^{2}\left[\rho \sum_{k=1}^{K}\sum_{\left(k,s\right)\in ℒ}\sum_{\left(k,t\right)\in ℒ}a_{\mathrm{ks}}^{\left(\pi \right)}\Gamma_{\mathrm{st}}a_{\mathrm{kt}}^{\left(\pi \right)}+\frac{4}{N}\left(1-\rho \right)\right].$$

Though any NA is optimal over its own support, it may, or may not, be optimal over the full layout ℒ. However, the main result given below shows that for any layout, ℒ, a NA for which $\mathrm{var}\;\hat{\theta }$ is a minimum (i.e., one which minimizes the summation term in (iii) above) is also an overall optimal allocation whenever the ICV falls below a threshold value which depends only on the CAC‐matrix.

### 4.2 The Main Result

For any layout ℒ with K cluster‐sequences and a fixed cluster‐autocorrelation matrix Γ, there is a threshold‐value $S_{1}\geq 1/\left(K+1\right)$, and a Natural Allocation, $\pi^{\left(0\right)}$—the “Best Natural Allocation”—such that $\pi^{\left(0\right)}$ is an optimal allocation over ℒ if and only if the

$$\mathrm{ICV}\leq S_{1}.$$

The result is proved in Appendix C.

When Γ is of full rank, $\pi^{\left(0\right)}$, is unique, and this is the usual situation. If not, any such NA will be optimal when the ICV $\leq S_{1}$.

If the average number of observations per cluster is fixed and equal to M (=N/K), it follows from the result above (subject to the usual caveats about non‐integer values) that there is a design, $n^{\left(0\right)}=N\pi^{\left(0\right)}$ which is optimal whenever the ICC, ρ, satisfies

(7) $$\rho \leq \frac{S_{1}}{\left(1-S_{1}\right)M+S_{1}}.$$

Moreover, this design has a natural treatment‐effect estimator for all values of ρ—that is even when it is not optimal.

Two simple examples are provided by multi‐period parallel and cross‐over layouts with K=2 cluster sequences and T time epochs with treatment assignments given by

$$Z_{\mathrm{kt}}=\left\{\begin{array}{ll} \frac{{\left(-1\right)}^{k+1}+1}{2} & \left(\text{parallel layout}\right)\\ \frac{{\left(-1\right)}^{k+t}+1}{2} & \;\left(\text{cross}-\text{over layout}\right) \end{array}\right.,$$

for k=1,2;t=1,…,T. Under a discrete‐time decay model with $\Gamma_{\mathrm{st}}=r^{\left|s-t\right|}$ it is shown in Appendix D that the best NA is optimal for all values of the ICV—so that $S_{1}=1$ in both examples—and takes the form

$$\pi_{11}^{\left(0\right)}=\pi_{12}^{\left(0\right)}=\pi_{T1}^{\left(0\right)}=\pi_{T2}^{\left(0\right)}=\frac{1}{2\left(2+\left(T-2\right)\left(1-u\right)\right)},$$

$$\pi_{1t}^{\left(0\right)}=\pi_{2t}^{\left(0\right)}=\frac{1-u}{2\left(2+\left(T-2\right)\left(1-u\right)\right)},\text{for}\;t=2,\ldots ,T-1$$

with u=+r for the parallel layout and u=−r for the cross‐over layout. This implies a uniform allocation over the interior time‐points (2≤t≤T−1) with some over‐ or under‐sampling at the end‐points according as the layout corresponds to a parallel or cross‐over design.

## 5 Optimal Allocations for Stepped‐Wedge Layouts

The results of Section 4 apply to any layout where there is at least one time‐epoch at which both Treated and Control cluster‐periods are present. From here on we focus on optimal allocations for a standard stepped‐wedge layout—where ℒ is a complete K×T lattice with T=K+1, and $Z_{\mathrm{kt}}=I_{\left\{t>k\right\}}$ for all (k,t)∈ℒ. Since Γ is assumed to be a Toeplitz matrix, it follows that such layouts, and their associated mixed models, are invariant under a transformation which combines time‐reversal (t→T−t+1) with interchange of treatment status $\left(Z_{\mathrm{kt}}\to 1-Z_{\mathrm{kt}}\right)$ and reverse‐ordering of cluster‐sequences (k→K−k+1). Consequently, any optimal allocation over a stepped‐wedge layout will be skew‐symmetric (in the sense of Bowden [20]) if it is unique or, at least, that a skew‐symmetric version of the optimal allocation must exist. Non‐uniqueness can occur only under the EXC version of our sampling model, for which the CAC‐matrix is not invertible.

### 5.1 Examples of Best Natural Allocations

**FIGURE 2:**
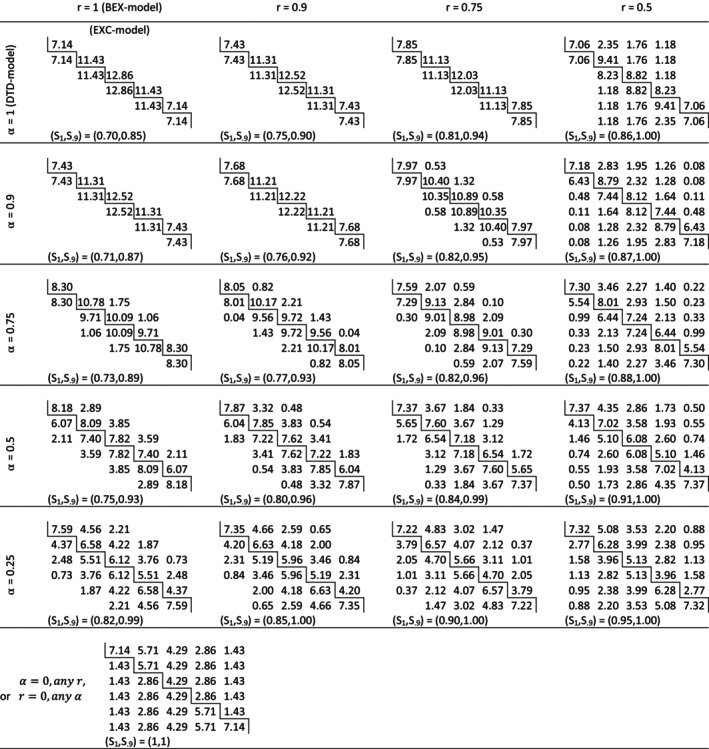
Best Natural Allocations for K = 6 clusters under cluster correlation models with $\Gamma_{\mathrm{st}}=\alpha r^{\left|s-t\right|}+\left(1-\alpha \right)\delta_{\mathrm{st}}$. For given α and r, the matrix‐entries are the percentage of the total number of observations allocated to each cluster at time periods 2 to 6. The solid lines represent the boundary between control and treated cells (as in Figure 1). The baseline (t=1) and endline (t=7) time‐periods are omitted because they do not feature in any best natural allocation. In each case, S_1_ and S_.9_ are the upper bounds for the ICV, S, below which the best natural design has 100% efficiency, and at least 90% efficiency, respectively. The bottom row contains the best natural allocation for a model with independent cluster‐periods.

Some best natural allocations for K=6 clusters in a stepped‐wedge layout were obtained numerically from the algorithm in Section 3 and are shown in Figure 2. For the models at the top‐left of the figure with (α,r)= (1), (1,0.9), (1,0.75), (0.9,1) or (0.9,0.9) the best natural allocations are ‘staircase’ solutions, by which we mean that their support is confined to cluster‐periods immediately before and immediately after a treatment switch, but excluding baseline and endline periods. As either α or r decrease away from 1, the support of the best natural allocation expands gradually into adjacent cells until the entire central region with 2≤t≤6 is occupied. In each case, the value of $S_{1}$ was estimated as largest value of the ICV for which the efficiency $\mathrm{Eff}\left(\pi^{\left(0\right)}\right)$ of the best natural allocation is at least 99.99%. It is shown alongside the threshold for 90% efficiency of the best natural allocation, denoted by $S_{.9}$. The minimum values of $S_{1}$ (= 0.70) and of $S_{.9}$ (= 0.85) occur at (α,r)=(1,1). In this sense, the best natural allocation performs least well under the EXC‐model: that is it has the smallest the range of values for S over which it is, or is close to, the optimal allocation. Elsewhere these thresholds increase along the rows of Figure 2 (as r decreases) and down the columns (as α decreases).

**FIGURE 3:**
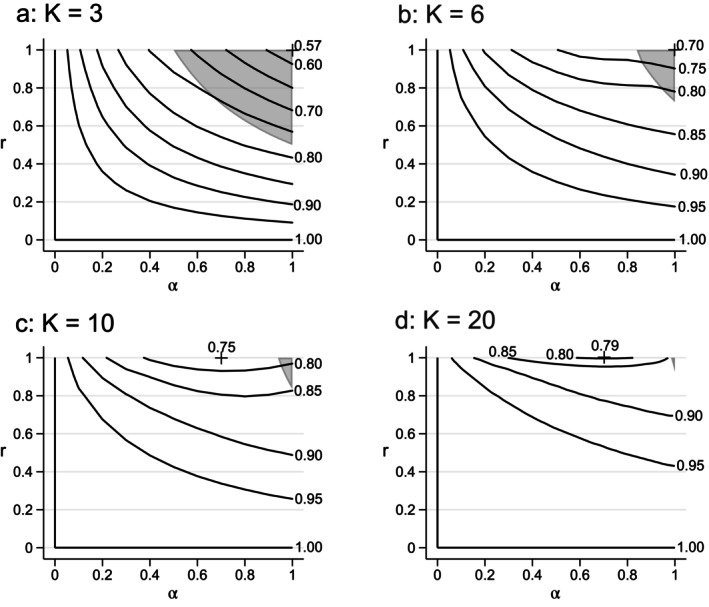
Contour‐plots for the ICV threshold value $S_{1}$ over the parameters (α,r) of the Γ‐family of cluster autocorrelation models. Contours are labeled with the values of $S_{1}$, and the minimum value indicated by a “+”. Within the shaded regions in the north‐east corners of the figures, the best natural allocation has a staircase support.

The contour‐plots in Figure 3 show how the value of $S_{1}$ varies with the cluster autocorrelation parameters α and r for four representative values of K. In general, it seems that $S_{1}\geq ½$ and increases with K, suggesting that best natural allocations may be more useful when the number of cluster‐sequences is larger. When K is small the minimum value of $S_{1}$ occurs for the EXC‐model, but for K≥8—specifically the values 7, 8, 9, 10 and 20 were investigated—the minimum occurs for a BEX‐model with r=1 and α close to 0.7.

Both the form of $\pi^{\left(0\right)}$ and the threshold value $S_{1}$ depend on the CAC‐matrix Γ and explicit solutions are available only in some special cases. For example, if either one of α or r=0 then Γ=I and observations are correlated only if they belong to the same cell of the layout. In this case there is an explicit expression for the best natural allocation (see Appendix E) namely.

(8) πkt(0)∝K−t+1ifk<tt−1ifk≥tfort=2,…,K,

corresponding to the allocation in the final row of Figure 2. Moreover, $\pi^{\left(0\right)}$ is fully optimal for all values of ρ and N (i.e., $S_{1}=1$). Here the BLUE of the treatment effect estimator has variance

(9) $$\mathrm{var}\;\hat{\theta }=\sigma^{2}\left[\frac{6}{K^{2}-1}\rho +\frac{4}{N}\left(1-\rho \right)\right].$$

If both α and r are non‐zero, we give explicit formulae in Section 6 for $\pi^{\left(0\right)}$ and $S_{1}$ in those cases where the best NA has a staircase support. But a complete solution to the optimisation problem—that is for any value of the ICV—has been obtained only under the EXC model (α=r=1) and is presented in Appendix H.

Though the Main Result simplifies the search for an optimal allocation when N and ρ are small enough that $S\leq S_{1}$, it has little to say about the solution in other cases. Nevertheless, it can be expected that the best natural allocation will be nearly optimal when S exceeds, but is close to, $S_{1}$.

### 5.2 Some Alternative Designs for the REaCH Study

The REaCH study comprised two stepped‐wedge cluster randomized trials in Nigeria and Tanzania [21]. The aim of the trials was to evaluate a training program for primary care clinics in low‐ and middle‐income countries to provide and improve access to remote (i.e., telephone) consulting services. Demand for such services increased during the Covid‐19 pandemic. Each trial included 20 clusters arranged as a double replicate of a standard stepped‐wedge design with K=10 sequences and 11 month‐long cluster‐periods.

Several primary outcomes—including the face‐to‐face and remote consultation rates—were measured using routinely collected data. In addition, a subset of patients who consulted each month were consented to participate in a study which included the PAM (“patient activation”) and PHBQ (“patient trust”) instruments, generating two scores assessing the quality of the visit. The initial design analysis proposed that 20 observations per cluster‐period should be collected in each replicate (i.e., K×(K+1)×20=2,200 in total) in order to achieve a 95% confidence interval of +/− 0.11 standard deviations for each score.

**FIGURE 4:**
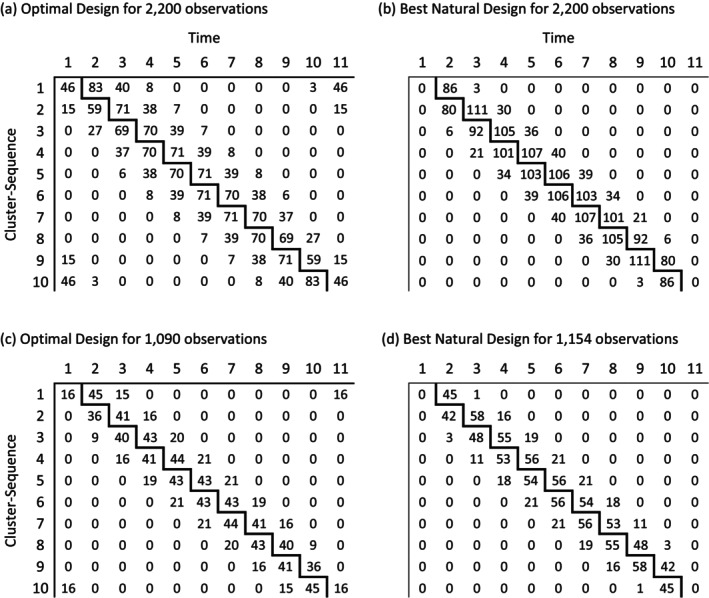
Designs associated with a standard stepped‐wedge layout with K = 10 cluster‐sequences and 20 observations per cell, that is 2200 in total, as used in the REaCH trial. Designs 4(a) and 4(b) are the Optimal and Best Natural designs for 2200 observations under a block‐exchangeable model with ICC = 0.05 and CAC = 0.8. These deliver greater precision than the standard layout. Design 4(c) is the Optimal Design for the least number of observations (= 1090) that has been chosen to achieve the same precision as the original standard layout. Design 4(d) is the Best Natural Design (with 1154 observations) that achieves this precision.

The patient activation and trust measures were time consuming and expensive to collect, yet the standard stepped‐wedge design employed here is relatively inefficient compared to the optimal design, under a range of plausible BEX‐models (Table 1 column 1). This suggests that savings might have been made by using some form of optimized design instead. Consider the case where ρ=0.05 and α=0.8. The 2‐replicate stepped‐wedge layout (as employed) leads to the required CI half‐width of 0.11 standard deviations, but is only 64% efficient compared to the optimal design in Figure 4a. Here the ICV is S=0.92, exceeding the threshold‐value $S_{1}$, which can be seen from Figure 3c to be less than 0.80. Thus the optimal design (Figure 4a) does not arise from a natural allocation. Although most of the observations are concentrated around the leading diagonal, some (about 6%) are isolated in the corners of the layout. We have found this to be a typical feature of optimal designs when the ICV exceeds the threshold value, $S_{1}$, and may be undesirable for logistical reasons. The best natural design in Figure 4b avoids this problem, though with some loss of precision—about 12% in this example. However, both designs are over‐powered for the requirements of the original study. These can be met more economically by employing optimal designs with many fewer observations, as in Figure 4c,d. Again, observations in the off‐diagonal corners of Figure 4c can be avoided by using a best natural allocation rather than a fully optimal one, though at the cost of 60 extra observations. Even so, the number of observations needed (1150) is only 52% of that used for the actual study. In the worst‐case scenario in Table 1 (i.e., as the CAC approaches 0) the number of observations needed to reproduce the stepped‐wedge precision (1320) is still only 60% of the original total.

**TABLE 1:** Improving the design of the REaCH study.

		Efficiency of the stepped‐ wedge design in the REaCH study (N=2200)	Total observations needed to achieve an equivalent precision using	
			(a) an optimal design	(b) a best natural design
ICC (ρ)	CAC (α)	%	N	N
0.05	1.0	41.62	880	880
0.05	0.8	63.95	1090	1150
0.05	0.5	76.10	1230	1290
0.05	0.2	81.63	1280	1280
0.05	0.0	80.51	1320	1320
0.01	1.0	43.97	940	940
0.01	0.8	52.63	1030	1030
0.01	0.5	60.30	1140	1140
0.01	0.2	65.87	1260	1260
0.01	0.0	66.72	1320	1320

*Note*: The efficiency of the Stepped‐wedge design used in the study is computed relative to the optimal design with the same number (2200) of observations under a BEX‐model. The numbers in the last two columns were obtained by reducing N from 2200 in units of 10 until further reduction would make the precision too low.

## 6 Optimal Staircase Designs

A major difficulty arises when seeking explicit forms for optimal allocations because the function Ψ(a) in (6) is not differentiable when the array a contains any zero components. Exploratory work with the algorithm over a stepped‐wedge layout, such as that leading to Figure 2, suggests that the set of non‐zero components often forms a staircase pattern. This is consistent with the work of Kasza et al. [10, 22], who showed that the information‐content of a complete stepped‐wedge design can be greatest in the staircase cells. Staircase solutions arise also from the combinatorial algorithm of Watson and Pan [13]. Here we proceed by first assuming a staircase pattern for the non‐zero components and then computing their optimal values by minimizing Ψ(a) using standard techniques. It then remains to verify that the resulting solution corresponds to a true minimum of Ψ(a) over the entire space of a‐values, or, at least to determine the conditions under which this is so. Full details are presented in Appendix G.

**FIGURE 5:**
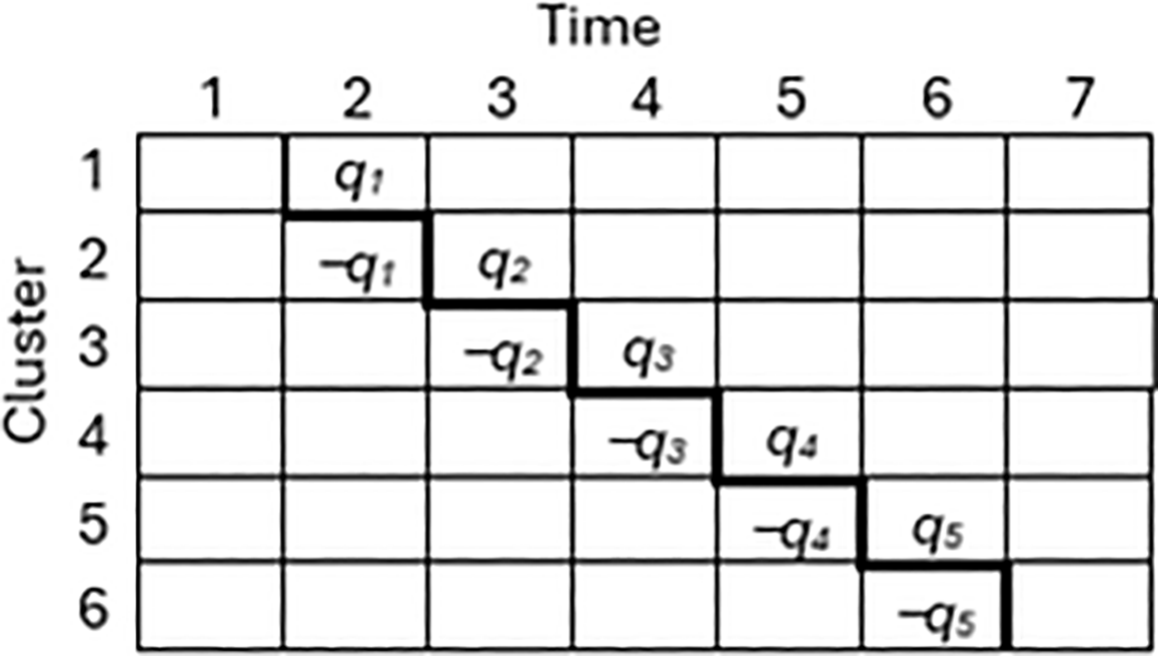
A staircase with BLUE coefficients for 6 clusters.

Formally, we define the ‘staircase’ support as the set of cells 𝒮={(t−1,t),(t,t);t=2,…,K}, and (tentatively) assume that it is the support of a best NA. Note that this definition differs from the basic staircase design of Grantham [23] only in that the points(1,1) and (K,K+1) are excluded. For ease of notation, we set the BLUE coefficients $a_{k,k+1}=q_{k}$ for k=1,…,K−1, and introduce two new quantities $q_{0}$ and $q_{K}$, both set equal to 0 here. Then $a_{\mathrm{kk}}=-q_{k}$ for k=2,…,K, as required by the constraints $\sum_{k}a_{\mathrm{kt}}=0$, (see Figure 5), and each $q_{k}>0$ because the design must have natural signs. Then $\sum_{\mathrm{kt}}\left|a_{\mathrm{kt}}\right|=2\sum_{k=1}^{K-1}q_{k}=2$ and the objective function (6) under the Γ‐family in (1) is

(10) $$\Psi \left(a\right)=\rho \sum_{k=0}^{K-1}\left(q_{k}^{2}-2\alpha rq_{k}q_{k+1}+q_{k+1}^{2}\right)+\frac{4}{N}\left(1-\rho \right).$$

The BLUE coefficients under the optimal staircase allocation are obtained in Appendix F by minimizing (10) subject to $\sum_{1}^{K-1}q_{k}=1$ using Lagrangian techniques. On setting $\phi =½\text{acosh}\left({\left(\alpha r\right)}^{-1}\right),$ this leads to.

(11) $${\tilde{q}}_{k}\propto \left\{\begin{array}{ll} \cosh\mathrm{K\phi}-\cosh\left(2k-K\right)\phi , & \text{if}\;\alpha r<1\\ k\left(K-k\right), & \text{if}\;\alpha r=1 \end{array}\right.$$

which holds over the full range of k‐values, including 0 and K, that is for k=0,…,K. The optimal staircase allocation is proportional to ${\tilde{q}}_{k}$ in each of the staircase cells—that is $\pi_{k,k+1}^{\left(0\right)}=\pi_{k+1,k+1}^{\left(0\right)}=½{\tilde{q}}_{k}$—and $\pi_{\mathrm{kt}}^{\left(0\right)}=0$ in all other cells. For fixed K, the allocation depends only on the first‐order cluster autocorrelation αr. The BLUE of the treatment effect θ has variance.

(12) $$\mathrm{var}\;\hat{\theta }=\left\{\begin{array}{ll} \left[\frac{2\left(1-\alpha r\right)\rho }{K-\coth\phi \tanh\mathrm{K\phi}}+\frac{4\left(1-\rho \right)}{N}\right]\sigma^{2}, & \text{if}\;\alpha r<1\\ \; & \\ \left[\frac{12\rho }{K\left(K^{2}-1\right)}+\frac{4\left(1-\rho \right)}{N}\right]\sigma^{2}, & \text{if}\;\alpha r=1 \end{array}\right.$$

It turns out that the staircase solution is the best natural allocation over the full stepped‐wedge layout whenever α and r are ‘sufficiently’ close to 1. In Appendix G we derive (a) the precise conditions under which this is so, together with (b) the upper threshold $S_{1}$ for the ICV which delineates the range of optimality for the staircase solution. These are as follows:

### 6.1 Conditions on the CAC Parameters for the Best Natural Allocation to Be a Staircase

If αr=1 the staircase solution is the best natural allocation. Otherwise (i.e., if αr<1), the staircase solution is the best natural allocation under the following conditions:

if K is an odd number (≥3) the condition is

(13) $$\frac{\cosh\mathrm{K\phi}}{\cosh\phi }\leq \frac{1-2\alpha r-\alpha r^{2}+2r}{1-2\alpha r+\alpha r^{2}};$$

if K is even (≥4) the condition is

(14) $$\cosh\mathrm{K\phi}\leq \frac{\min\left(2r-\alpha r-\alpha^{2}r^{3},1+\alpha r^{2}-2\alpha^{2}r^{2}\right)}{\alpha r\left(1-2\alpha r+\alpha r^{2}\right)}.$$

As before, ϕ is determined by the formula $\phi =½\text{acosh}\left({\left(\alpha r\right)}^{-1}\right)$, and the conditions express an upper bound on the number of cluster‐sequences, K, (Table 2) for which the best‐natural allocation is a staircase. If K is fixed, they specify a region in the (α,r)‐plane, in the North‐East corner of the unit square, as illustrated in the panels of Figure 3.

**TABLE 2:** Maximum number of clusters for which the base‐optimal allocation has staircase support when $\Gamma_{\mathrm{st}}=\alpha r^{\left|s-t\right|}+\left(1-\alpha \right)\delta_{\mathrm{st}}$.

		r							
		0.5	0.6	0.7	0.8	0.9	0.95	0.99	1.0 (BEX)
α	0.5	2	2	2	2	2	2	2	3
	0.6	2	2	2	2	3	3	3	3
	0.7	2	2	2	3	3	4	4	4
	0.8	2	2	3	4	4	4	5	5
	0.9	2	3	4	5	6	7	7	7
	0.95	2	3	4	6	8	9	10	10
	0.99	2	3	5	7	12	17	22	24
	1.0 (DTD)	3	3	5	8	15	27	84	∞ (EXC)

*Note*: The last row and last column correspond to the DTD and BEX models, respectively. Under the EXC model (α=r=1), the best natural allocation always has staircase support (i.e., maxK=∞).

### 6.2 The ICV Threshold

When the CAC conditions under (a) above are satisfied, the staircase solution is fully optimal if

(15) $$S\leq S_{1}=\frac{2}{2+K\left(1+r^{K-2}\right)\alpha r\pi_{12}^{\left(0\right)}},$$

where $\pi_{12}^{\left(0\right)}\;\left(=½{\tilde{q}}_{1}\right)$ is the proportion of the sample allocated to cluster 1 at time 2. An explicit form for ${\tilde{q}}_{k}$ can be found in Equation (F2) in Appendix F. Under the EXC‐model (α=r=1) we have ${\tilde{q}}_{1}=6/\left(K\left(K+1\right)\right)$ and $S_{1}=\left(K+1\right)/\left(K+4\right)$.

### 6.3 Staircase Optimality Under the BEX‐ and DTD‐Models

The Γ‐family of models in (1) includes the BEX‐ and DTD‐models, both of which have been proposed for the analysis of cluster‐studies. Under the BEX‐model (r=1), the conditions (13) and (14) reduce to:

$$\frac{\cosh\mathrm{K\phi}}{\cosh\phi }\leq 3,\text{if}\;K\;\text{is}\;\mathrm{odd},$$

and

$$\cosh\mathrm{K\phi}\leq 2+\frac{1}{\alpha },\text{if}\;K\;\text{is even},$$

with $\phi =½\text{acosh}\left(\alpha^{-1}\right).$

For the DTD‐model (α=1), they become:

$$\frac{\cosh\mathrm{K\phi}}{\cosh\phi }\leq \frac{1+r}{1-r}=\coth^{2}\phi ,\text{if}\;K\;\text{is}\;\mathrm{odd},$$

and

$$\cosh\mathrm{K\phi}\leq \frac{1+r}{1-r}=\coth^{2}\phi ,\text{if}\;K\;\text{is even},$$

with $\phi =½\text{acosh}\left(r^{-1}\right).$Under both models, the CAC conditions amount to a lower bound on the first‐order cluster autocorrelation (=α, or r). The staircase solution is optimal over a more extensive range of first‐order autocorrelations under the DTD‐model, though the range approaches a single point (i.e., 1) for both models as K increases (Table 3), and indeed for any model in the Γ‐family (witness the way the shaded regions become smaller over the four panels of Figure 3).

**TABLE 3:** Thresholds for the first‐order cluster autocorrelation (=αr) above which $\pi^{\left(0\right)}$ is a staircase, for the DTD‐ and BEX‐models. Bracketed entries show the corresponding threshold for the autocorrelation between the beginning and end of the study for the DTD‐model ($=r^{K})$.

K	3	4	6	8	10	15	20	30	50	100
DTD‐model (r‐threshold)	0.500	0.618	0.726	0.792	0.835	0.894	0.926	0.957	0.979	0.992
DTD‐model ($r^{K}$‐threshold)	(0.125)	(0.146)	(0.147)	(0.154)	(0.164)	(0.188)	(0.217)	(0.264)	(0.339)	(0.460)
BEX‐model (α – threshold)	0.500	0.667	0.839	0.907	0.939	0.976	0.986	0.993	0.998	0.999

In our formulation, an increase in the number of clusters (K) is automatically accompanied by an increase in the number of time‐epochs (T=K+1), implying a more refined categorization of time. In practice, the autocorrelation across the entire study‐length ($\Gamma_{1,K+1}=\alpha r^{K}$), as in Hooper et al. [14], may be a more meaningful representation of cluster‐level effects than the first order autocorrelation, αr, whose interpretation is sensitive to the time‐categorization. Under the DTD‐model the threshold for the study‐length autocorrelation does not rise dramatically with K: it is around 0.15 for small K, and remains less than 0.5 for all values of K up to 124 (Table 3). So, for realistic numbers of cluster‐sequences, the threshold will be exceeded—and the best natural allocation will be a staircase—unless the between‐period ICC reduces substantially over the duration of the study.

**FIGURE 6:**
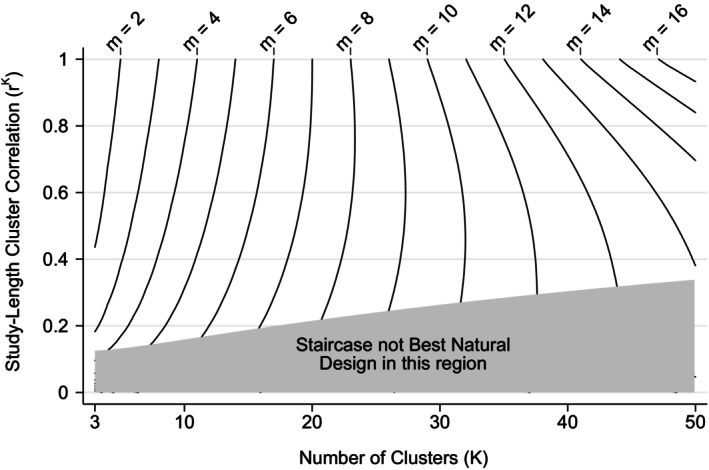
Contours of $S_{1}$, for best natural staircase allocations under the DTD‐model, as a function of K, the number of clusters, and $r^{K}$, the study‐length cluster autocorrelation. The contours are labeled by $m=S_{1}/\left(1-S_{1}\right)$. Outside the excluded (gray) region, the optimal design is a staircase if the ICC does not exceed m/(m+M), where M=N/K is the average number of observations per cluster.

A best natural (staircase) allocation may not be the optimal allocation, because of the ICV condition. The situation is summarized in Figure 6 for the DTD‐model. The best natural allocation is a staircase for all points above the excluded region. The contours refer to the threshold values $S_{1}$, and have been chosen so that the ratio $S_{1}/\left(1-S_{1}\right)$ takes an integer value =m, say. On such a contour the (best‐natural) staircase solution is fully optimal provided that the ICC satisfies.

(16) $$\rho \leq \frac{m}{m+M}\;\text{or},\text{equivalently},M\leq m\frac{1-\rho }{\rho }.$$

In every case $S_{1}\geq 1/2$ and m≥1. This implies that the optimal design can be a staircase even if the number of clusters is small—provided that the ICC is less than 1/(1+M) and the study‐length autocorrelation is large enough to miss the excluded region in Figure 6. Larger values of the ICC can lead to a staircase optimum if the clusters are small (i.e., small M), or the number of clusters (K) is large. When r=1 (the EXC‐model) we have $S_{1}=\left(K+1\right)/\left(K+4\right)$, m=(K+1)/3 and the optimal design is a staircase if ρ≤(K+1)/(K+1+3M).

Korevaar [24] has fitted the DTD, EXC and BEX models to 44 continuous outcomes from 29 longitudinal studies with three different types of cluster‐design. Under the DTD‐model they found the median ρ = 0.05 (quartiles 0.02, 0.09), and the median r=0.73 (0.19, 0.91). In 4 of their 7 stepped‐wedge examples the study‐length cluster autocorrelation, $r^{T-1}$, ranged from 0.33 to 0.83, with median 0.69, but was close to 0 (i.e., ≤0.05) in the other 3 examples. Thus, the best natural allocation would have been a staircase in 4 out of 7 stepped‐wedge studies—given that the number of clusters is to be no more than 50 (Table 3, Figure 6). Taking 0.66 as a typical value for this autocorrelation in such studies and using a range of values for ρ=0.01,0.05,0.10,0.25, Table 4 shows the maximum average cluster‐size, M, for which the staircase would have been the overall optimal design, from (16). As expected, the maximum cluster‐size increases with K but is decreasing in ρ.

**TABLE 4:** Upper limit on average cluster size (M) under which the optimal design is a staircase, assuming a DTD model with study‐length autocorrelation $r^{K}=0.66$.

ICC, *ρ*	K = 6	K = 10	K = 20	K = 30	K = 50
0.01	274	411	706	956	1368
0.05	53	79	136	183	263
0.10	25	37	64	87	124
0.25	8	12	21	29	41

**FIGURE 7:**
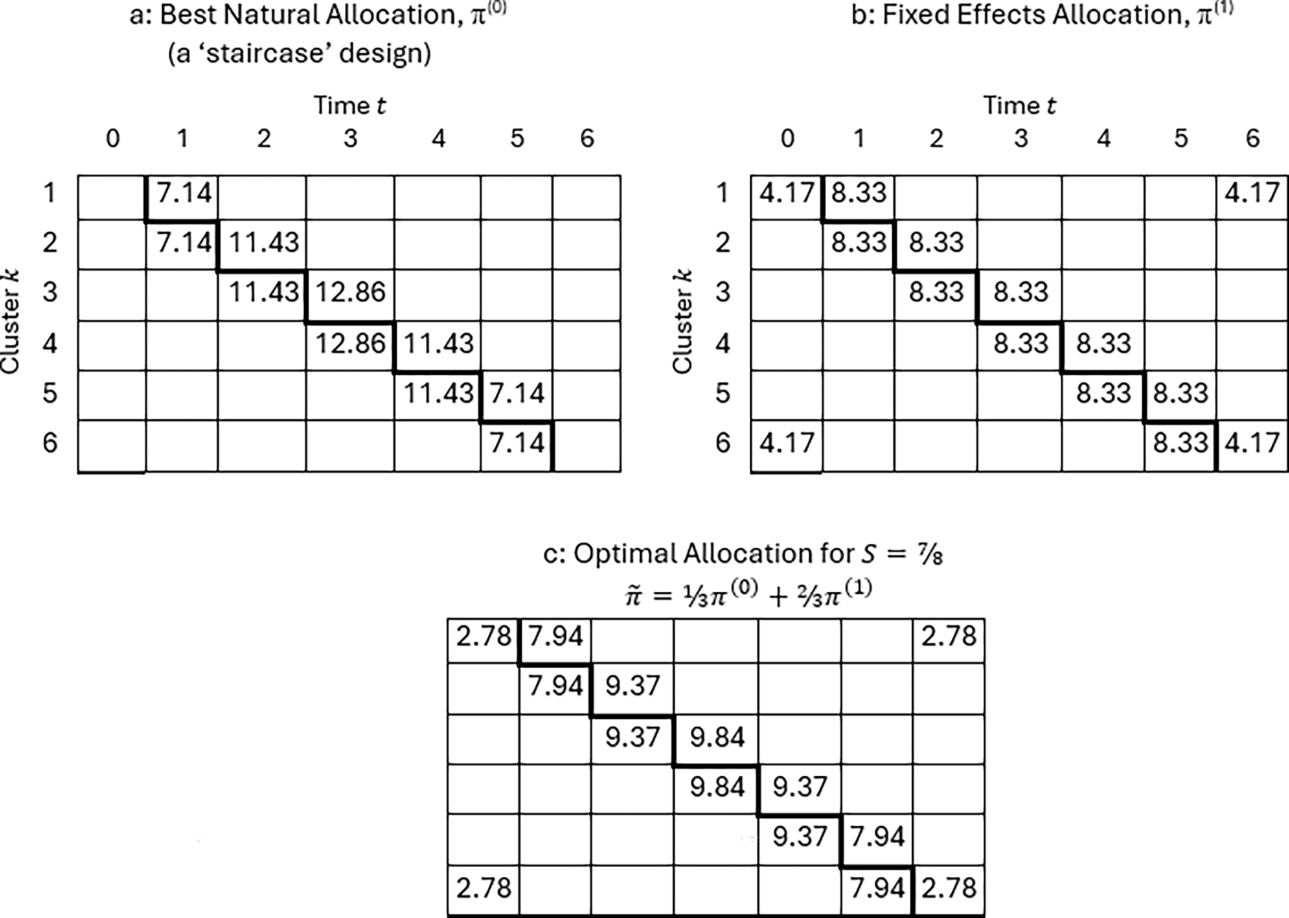
Optimal allocations for K=6 clusters under an EXC‐model with ICV S=7/8, expressed as percentages of the total number of observations. Cells with 0% have been left blank. The allocation in Figure 7c is a weighted combination of Figure 7a and Figure 7b with weights ⅓ [=⅓(K+1)(1−S)/S] and ⅔.

The efficiency of the staircase design is sometimes quite high even when it (the staircase) is not fully optimal, so that larger cluster‐sizes may lead to satisfactory performance in practice. An example of a fully optimal allocation when $S>S_{1}$ under the EXC model is shown in Figure 7c. It consists of a weighted average of the best natural (staircase) allocation (Figure 7a) with an allocation that is optimal as ρ→1 (Figure 7b) and includes ‘hotspots’ in the corners of the layout—such as arise also in the work of Kasza [10, 22] and of Hooper [14]. Exact details are given in Appendix H. Compared to the optimal allocation, the best natural staircase is 84% efficient. Hooper et al. [14] remark on the high relative efficiency (> 95%) of staircase‐type designs over all considered scenarios. Their scenarios have K=30 clusters and study‐length autocorrelations of 0.2 and 0.04, which both fall below the threshold for a best natural staircase allocation, the latter emphatically so (Table 3). Several of their optimal designs have a suggestive staircase structure, though their results are not strictly comparable with ours, not least because of their use of a low‐order polynomial time‐effect.

## 7 Discussion

We have presented some exact results for an optimal design problem for cluster studies within a linear mixed effects model in discrete time, together with an algorithm for finding optimal designs. The aim is to distribute a fixed number of observations over an experimental layout consisting of a pre‐specified set of cluster‐sequences, with a particular focus on stepped‐wedge layouts. The formulation applies to cross‐sectional cluster studies where a fresh set of subjects is available at each new time epoch. For convenience, we have assumed that a single cluster will be randomized to each cluster‐sequence in the resulting design, but the results also apply where this design is replicated over several equal groups of clusters.

In many conventional designs (e.g., parallel and balanced cross‐over designs) the BLUE of the treatment‐effect can be expressed as the difference between the means of the treated and control observations. This is not the case for standard stepped‐wedge designs where observations from different cluster‐periods will have different coefficients and some will even present with counter‐intuitive signs, seemingly at variance with their treatment status. This may be seen as a problem for the interpretation of experimental data. The best natural allocation overcomes this problem by insisting that the treatment‐effect estimate has a natural interpretation. Moreover, the best natural allocation actually solves the optimization problem in cases where the ICV is less than the threshold value, $S_{1}$, as will happen when the total number of observations and the ICC are relatively small. For stepped‐wedge layouts it seems that the best natural allocation requires that each cluster is sampled only in consecutive time‐periods; or, at least, we have yet to find an instance where this is not the case. On the other hand, an allocation that is exactly optimal for a larger ICV‐value often entails observations from “hotspots” in the corners of the design, which are separated in time from the main sampling effort. Under such a design, some clusters could experience a lengthy interruption in data collection, with possible attendant logistical problems. Sometimes this will be too high a price to pay for the improvement in precision over a best natural design, which might then be preferred even when the ICV exceeds the threshold value. The precise form of the best natural allocation depends on the cluster autocorrelations (in Γ), which are generally less accessible than the value of the ICC. Under the EXC‐model these autocorrelations are pre‐specified ($\Gamma_{\mathrm{st}}\equiv 1$) even at the analysis stage, and the best natural allocation will lead to a treatment‐effect estimate which is a simple difference between treated and control mean values—an outcome with obvious appeal. For other models, the components of Γ, though assumed known for design purposes, would likely be regarded as unknown at the analysis stage, with the consequential effect that the treatment‐effect estimate might not take such a simple form. One may expect that it will be ‘close’ to the mean‐difference estimate unless the presumed form for Γ is badly misspecified, but we have not investigated this issue.

For stepped‐wedge layouts we present precise conditions under which the optimal allocation has a staircase structure, under a parametric model for the cluster‐period correlation structure which includes the Exchangeable, Block‐Exchangeable and Discrete‐time Decay models as special cases. This builds on the work of Kasza [10, 22], who noted that the information content in a stepped‐wedge design is often concentrated in the staircase cells, and may provide some theoretical underpinning for the staircase designs that emerge from other algorithmic approaches. However, optimality in our designs is achieved by varying the size of the samples in the stepped‐wedge cells on a continuous scale, whereas some other approaches [5, 11] work with cells of fixed size which may or may not be included in the final design. Similarly, our staircase designs do not precisely coincide with those considered by Grantham [23], where the number of observations in each cluster‐period is fixed. In our formulation, an optimal staircase design emerges under sampling schemes for which the correlation between observations in the same cluster is relatively low, and decreases slowly, or not at all, as the interval between them increases—that is, a small ICC combined with large CACs. Under the DTD model, the CAC condition depends only on the rate of decay and the number of cluster‐sequences. If this is less than 50 the condition certainly holds when the study‐length CAC exceeds ½. The requirements on the ICC are more complex since they vary also with the overall sample size. However, they will often be satisfied in studies where the clusters are small.

We have modeled time as divided into discrete epochs, defined by the instants at which the cluster‐sequences receive the treatment on the first occasion, with a separate time effect for each epoch. Under a stepped‐wedge layout, the number of time epochs is determined effectively by the number of cluster‐sequences under consideration—a somewhat arbitrary constraint, though one that is commonly adopted in the analysis of such studies. Here it has the consequence that treated observations are accompanied by an equal number of contemporaneous controls in any optimal design. A drawback is that the implied length of the epochs in real time is entangled with the model for the cluster‐correlation structure. For example, where recruitment rates are limited, it may be difficult to achieve the number of observations required in certain cells of an optimal design without increasing the length of the cluster periods in real time. This would increase the overall duration of the study and entail a modification of the cluster‐period correlation structure which, in some cases, might impact the form of the optimal design. One suggestion is to increase the number of clusters and reduce the number of observations per cluster so as to achieve the same target precision. Where this is possible, it would likely alleviate some problems associated with limited recruitment rates within individual clusters. Otherwise, a move away from discrete time models points to the need for a better understanding of the optimal design problem in continuous time [25], where both the timing of the switch points and the sampling schedule within clusters can be chosen at will. Our results entail a complete solution to this problem only under the exchangeable model, where the passage of time has no effect on the correlation between observations.

Our approach follows a common strategy in the optimal design literature of identifying the optimal design in terms of a probability measure over a design space [26]. In practical applications, one must translate the masses this measure ($\tilde{\pi }$) places on each design point into integer numbers of observations ($n_{\mathrm{kt}}$). Pukelsheim and Rieder [18] provide a discussion of rounding weights in this context. Usually, the proportions $\tilde{\pi }$ do not often provide enough information to uniquely identify an optimal design n. Many rounding procedures exist, but the number of resulting designs is often small enough to directly calculate the power of each and identify the most efficient. In practice, the differences in efficiency are likely to be minimal, particularly for larger sample sizes.

## 8 Conclusions

The focus on the best natural allocation and the index of cluster variation clarifies the contributions of the different types of correlation parameters in determining the optimal allocation of observations among the cells of an experimental layout. Consideration should be given to using best natural allocations for generating study designs in practice. Further investigation of the efficiency of such allocations when the conditions for their optimality are violated could have important implications for study design.

## Conflicts of Interest

The authors declare no conflicts of interest.

## Supporting information


**Data S1:** Supporting Information.

## Data Availability

Data sharing not applicable to this article as no datasets were generated or analysed during the current study.

## Appendix A Mathematical Arguments

The set of feasible arrays of BLUE coefficients on ℒ is denoted by

$$𝒜=\left\{a;\sum_{\underset{\left(k,t\right)\in ℒ}{k=1}}^{K}a_{\mathrm{kt}}=0\;\text{for}\;\mathrm{all}\;t=1,\ldots ,T,\sum_{\left(k,t\right)\in ℒ}a_{\mathrm{kt}}Z_{\mathrm{kt}}=1\right\}.$$

Define the functions $F,H,G_{\pi }:𝒜\to \mathbb{R}$ by

$$F\left(a\right)=\sum_{k}\sum_{\underset{\left(k,s\right)\in ℒ}{s}}\sum_{\underset{\left(k,t\right)\in ℒ}{t}}a_{\mathrm{ks}}\Gamma_{\mathrm{st}}a_{\mathrm{kt}},H\left(a\right)={\left(\underset{\left(k,t\right)\in ℒ}{\sum_{k}\sum_{t}\;}\left|a_{\mathrm{kt}}\right|\right)}^{2},$$

and

$$G_{\pi }\left(a\right)=\underset{\left(k,t\right)\in 𝒫\left(\pi \right)}{\sum_{k}\sum_{t}\;}\frac{a_{\mathrm{kt}}^{2}}{\pi_{\mathrm{kt}}}.$$

where π is any allocation on ℒ. Also define 𝒫(π)=supp(π), the support of π, and let.

${𝒜}_{\pi }=𝒜\cap \left\{a;\mathrm{supp}\left(a\right)\subset 𝒫\left(\pi \right)\right\},$ the set of a∈𝒜 whose components are zero outside the support of π.

With these definitions, we can write (4) and (6) as

$$V\left(a,N\pi \right)=\sigma^{2}\left[\rho F\left(a\right)+\frac{\left(1-\rho \right)}{N}G_{\pi }\left(a\right)\right]$$

and

$$\Psi \left(a\right)=\rho F\left(a\right)+\frac{1-\rho }{N}H\left(a\right).$$

### Equations (5) and (6)

Suppose that π is some allocation on ℒ—that is $\pi_{\mathrm{kt}}\geq 0$ for (k,t)∈ℒ and $\sum_{ℒ}\pi_{\mathrm{kt}}=1$. By the Cauchy‐Schwarz inequality we have, for $a\in {𝒜}_{\pi }$,

(A1)
$$H\left(a\right)={\left(\sum_{\left(k,t\right)\in 𝒫\left(\pi \right)}\frac{\left|a_{\mathrm{kt}}\right|}{\sqrt{\pi_{\mathrm{kt}}}}\cdot \sqrt{\pi_{\mathrm{kt}}}\right)}^{2}\leq \sum_{𝒫\left(\pi \right)}\frac{{\left|a_{\mathrm{kt}}\right|}^{2}}{\pi_{\mathrm{kt}}}\cdot \sum_{𝒫\left(\pi \right)}\pi_{\mathrm{kt}}=\sum_{𝒫\left(\pi \right)}\frac{a_{\mathrm{kt}}^{2}}{\pi_{\mathrm{kt}}}=G_{\pi }\left(a\right)$$

with equality if and only if $\sqrt{\pi_{\mathrm{kt}}}\propto \left|a_{\mathrm{kt}}\right|/\sqrt{\pi_{\mathrm{kt}}}$ on 𝒫(π). So, if a∈𝒜 is fixed and π is constrained so that 𝒫(π)⊃supp(a), it follows that $G_{\pi }\left(a\right)$ is minimized as a function of π when $\pi_{\mathrm{kt}}=\left|a_{\mathrm{kt}}\right|/\sum_{\left(l,s\right)\in ℒ}\left|a_{\mathrm{ls}}\right|$, which is equation (5).

At the minimum we have

$$G_{\pi }\left(a\right)=\sum_{\left(k,t\right)\in 𝒫\left(\pi \right)}\frac{a_{\mathrm{kt}}^{2}}{\pi_{\mathrm{kt}}}=\sum_{\left(k,t\right)\in 𝒫\left(\pi \right)}a_{\mathrm{kt}}^{2}\frac{\sum_{ℒ}\left|a_{\mathrm{ls}}\right|}{\left|a_{\mathrm{kt}}\right|}$$

$$\;=\sum_{\left(k,t\right)\in 𝒫\left(\pi \right)}\left|a_{\mathrm{kt}}\right|\sum_{\left(l,s\right)\in ℒ}\left|a_{\mathrm{ls}}\right|={\left(\sum_{\left(k,t\right)\in ℒ}\left|a_{\mathrm{kt}}\right|\right)}^{2}=H\left(a\right),$$

because $a_{\mathrm{kt}}=0$ when (k,t)∉𝒫(π). Then it follows from (4) that $\Psi \left(a\right)=\rho F\left(a\right)+\frac{1-\rho }{N}H\left(a\right)$, which is Equation (6). ■

## Appendix B Properties of Natural Allocations (NAs)

- Whether π is a NA or not depends on the cluster autocorrelation matrix, Γ, but is independent of the total sample size N and the ICC, ρ. In particular, it is independent of the ICV, S.

Recall that $Z_{\mathrm{kt}}\left(=0,1\right)$ denotes the treatment status of cell (k,t). Then for a∈𝒜, we have

$$\sqrt{H\left(a\right)}=\sum_{\left(k,t\right)\in ℒ}\left|a_{\mathrm{kt}}\right|=\sum_{ℒ}\left[\left|a_{\mathrm{kt}}\right|Z_{\mathrm{kt}}+\left|a_{\mathrm{kt}}\right|\left(1-Z_{\mathrm{kt}}\right)\right]$$

$$=\sum_{ℒ}\left|a_{\mathrm{kt}}Z_{\mathrm{kt}}\right|+\sum_{ℒ}\left|a_{\mathrm{kt}}\left(1-Z_{\mathrm{kt}}\right)\right|\geq \left|\sum_{ℒ}a_{\mathrm{kt}}Z_{\mathrm{kt}}\right|+\left|\sum_{ℒ}\left(a_{\mathrm{kt}}-a_{\mathrm{kt}}Z_{\mathrm{kt}}\right)\right|$$

$$=\left|\sum_{ℒ}a_{\mathrm{kt}}Z_{\mathrm{kt}}\right|+\left|0-\sum_{ℒ}a_{\mathrm{kt}}Z_{\mathrm{kt}}\right|=2\left|\sum_{ℒ}a_{\mathrm{kt}}Z_{\mathrm{kt}}\right|=2.$$

This result, together with that of (A1) above, shows that

(B1)
$$G_{\pi }\left(a\right)\geq H\left(a\right)\geq 4\;\text{for}\;\mathrm{any}\;a\in {𝒜}_{\pi }.$$

Now let λ=Nρ/(1−ρ)=KS/(1−S). Then π is a NA if and only if $a=a^{\left(\pi \right)}=2\left(2Z_{\mathrm{kt}}-1\right)\pi_{\mathrm{kt}}$ minimizes

$$\lambda F\left(a\right)+G_{\pi }\left(a\right)$$

over $a\in {𝒜}_{\pi }$. Now

$$G_{\pi }\left(a^{\left(\pi \right)}\right)=\sum_{\left(k,t\right)\in 𝒫\left(\pi \right)}4\pi_{\mathrm{kt}}^{2}/\pi_{\mathrm{kt}}=4\sum_{𝒫\left(\pi \right)}\pi_{\mathrm{kt}}=4.$$

From (B1) this means that $a=a^{\left(\pi \right)}$ minimizes $G_{\pi }\left(a\right)$ over ${𝒜}_{\pi }$. The functions F and $G_{\pi }$ are both convex and differentiable over ${𝒜}_{\pi }$, with zero derivatives at their minima. Hence π will be an NA if and only if $a=a^{\left(\pi \right)}$ also minimizes F(a) over ${𝒜}_{\pi }$. This condition does not involve the quantity λ. Whether π is a NA depends only on the cluster autocorrelation matrix, Γ, but not on N or ρ, as claimed.

ii If π is a NA, it is the optimal allocation over its own support—that is over the sub‐layout $𝒫\left(\pi \right)=\mathrm{supp}\left(\pi \right)=\left\{\left(k,t\right)\in ℒ;\pi_{\mathrm{kt}}>0\right\}$, for all values of N and ρ.

From (B1), H(a) also achieves its minimum value of 4 at $a=a^{\left(\pi \right)}$. It follows that $a^{\left(\pi \right)}$ also minimizes

λF(a)+H(a)

over ${𝒜}_{\pi }$ whenever π is a NA. In this case the optimal allocation satisfies

$${\tilde{\pi }}_{\mathrm{kt}}=\frac{\left|a_{\mathrm{kt}}^{\left(\pi \right)}\right|}{\sum_{𝒫\left(\pi \right)}\left|a_{\mathrm{ls}}^{\left(\pi \right)}\right|}=\frac{2\pi_{\mathrm{kt}}}{2\sum_{𝒫\left(\pi \right)}\pi_{\mathrm{ls}}}=\pi_{\mathrm{kt}},\text{from}\;\left(5\right).$$

In other words, if π is a NA, it is the optimal allocation over 𝒫(π) as claimed.

iii If π is a NA the treatment effect estimator has variance
  $$\mathrm{var}\;\hat{\theta }=\sigma^{2}\left[\rho \sum_{k=1}^{K}\sum_{\left(k,s\right)\in ℒ}\sum_{\left(k,t\right)\in ℒ}a_{\mathrm{ks}}^{\left(\pi \right)}\Gamma_{\mathrm{st}}a_{\mathrm{kt}}^{\left(\pi \right)}+\frac{4}{N}\left(1-\rho \right)\right],$$

where $a_{\mathrm{kt}}^{\left(\pi \right)}=2\left(2Z_{\mathrm{kt}}-1\right)\pi_{\mathrm{kt}}$. Using (4), we have

(B2)
$$\begin{array}{cc} \mathrm{var}\;\hat{\theta } & =\frac{\sigma^{2}\left(1-\rho \right)}{N}\left[\lambda F\left(a^{\left(\pi \right)}\right)+G_{\pi }\left(a^{\left(\pi \right)}\right)\right]\\ & \;=\sigma^{2}\left[\rho \sum_{k=1}^{K}\sum_{s}\sum_{t}a_{\mathrm{ks}}^{\left(\pi \right)}\Gamma_{\mathrm{st}}a_{\mathrm{kt}}^{\left(\pi \right)}+\frac{4}{N}\left(1-\rho \right)\right], \end{array}$$

as claimed, since $G_{\pi }\left(a^{\left(\pi \right)}\right)=4$.

## Appendix C The Main Result

For any layout ℒ with K cluster‐sequences and a fixed cluster‐autocorrelation matrix Γ, there is a threshold‐value $S_{1}\geq 1/\left(K+1\right)$, and a Natural Allocation, $\pi^{\left(0\right)}$—the “best natural allocation”—such that $\pi^{\left(0\right)}$ is an optimal allocation over ℒ if and only if the ICV $\leq S_{1}$. Throughout this proof it is to be understood that $a_{\mathrm{kt}}$ is defined only for (k,t)∈ℒ.

- First, we show that the optimal allocation is a NA whenever S≤1/(K+1).

From (6), the optimal BLUE coefficients minimize

Φ(a)=λF(a)+H(a),overa∈𝒜,

where λ=Nρ/(1−ρ)=KS/(1−S). The condition S≤1/(K+1) translates toλ≤1. Assuming λ≤1, we will show that the BLUE coefficients are naturally signed – that is that $a_{\mathrm{kt}}\left(2Z_{\mathrm{kt}}-1\right)\geq 0$ for all (k,t)∈ℒ. Suppose that a∈𝒜 is not naturally signed, with $a_{\mathrm{kt}}<0$ for some treated cell (k,t) – that is with $Z_{\mathrm{kt}}=1$. We construct an array of coefficients $a^{\prime }\in 𝒜$ for which $\Phi \left(a^{\prime }\right)<\Phi \left(a\right),$ so that a cannot be optimal. A similar argument shows that acannot be optimal if $a_{\mathrm{kt}}>0$ for some control cell. (In the following paragraphs the restriction that $a_{\mathrm{kt}}$ is defined only for (k,t)∈ℒ is to be understood, but is sometimes suppressed to avoid notational clutter.) Since $\sum_{\left\{i;\left(i,t\right)\in ℒ\right\}}a_{\mathrm{it}}=0$, there must be a cell (l,t) for which $a_{\mathrm{lt}}>0$. For some (small) ε>0 define

$$b_{\mathrm{kt}}=a_{\mathrm{kt}}+\varepsilon ;b_{\mathrm{lt}}=a_{\mathrm{lt}}-\varepsilon ;b_{\mathrm{ju}}=a_{\mathrm{ju}}\;\text{for}\;\left(j,u\right)\neq \left(k,t\right)\;\text{or}\;\left(l,t\right),$$

and set

$$a_{\text{is}}^{\prime }=\frac{b_{\text{is}}}{\sum_{\mathrm{ju}}b_{\mathrm{ju}}Z_{\mathrm{ju}}}\;\text{for}\;\left(i,s\right)\in ℒ.$$

Clearly $\sum_{i}a_{\text{is}}^{\prime }=0$ for all s and $\sum_{\text{is}}a_{\text{is}}^{\prime }Z_{\text{is}}=1$ so that $a^{\prime }\in 𝒜$. Also $\sum_{\mathrm{ju}}\left|b_{\mathrm{ju}}\right|=\sum_{\mathrm{ju}}\left|a_{\mathrm{ju}}\right|-2\varepsilon$, and $\sum_{\mathrm{ju}}b_{\mathrm{ju}}Z_{\mathrm{ju}}=1,\text{or}\;1+\varepsilon$, according as (l,t) is, or is not, a treated cell. Then we have

$$\begin{array}{cc} & {\left(\sum_{\mathrm{ju}}b_{\mathrm{ju}}Z_{\mathrm{ju}}\right)}^{2}\Phi \left(a^{\prime }\right)=\Phi \left(b\right)=\Phi \left(a\right)+2\lambda \varepsilon \sum_{s=1}^{T}\Gamma_{\mathrm{ts}}\left(a_{\mathrm{ks}}-a_{\mathrm{ls}}\right)\\ & \;-4\varepsilon \sum_{\mathrm{ju}}\left|a_{\mathrm{ju}}\right|+o\left(\left\|\varepsilon \right\|\right). \end{array}$$

But $\left|\sum_{s}\Gamma_{\mathrm{ts}}\left(a_{\mathrm{ks}}-a_{\mathrm{ls}}\right)\right|\leq \sum_{s}\left|\Gamma_{\mathrm{ts}}\right|\left|a_{\mathrm{ks}}-a_{\mathrm{ls}}\right|\leq \sum_{s}\left|a_{\mathrm{ks}}-a_{\mathrm{ls}}\right|\leq 2\sum_{\mathrm{ju}}\left|a_{\mathrm{ju}}\right|$, and ${\left(\sum_{\mathrm{ju}}b_{\mathrm{ju}}Z_{\mathrm{ju}}\right)}^{-2}\leq {\left(1+\varepsilon \right)}^{-2}=1-2\varepsilon +o\left(\left|\left|\varepsilon \right|\right|\right)$. Hence

$$\Phi \left(a^{\prime }\right)\leq \Phi \left(a\right)+2\varepsilon \left[2\left(\lambda -1\right)\sum_{\mathrm{ju}}\left|a_{\mathrm{ju}}\right|-\Phi \left(a\right)\right]+o\left(\left\|\varepsilon \right\|\right).$$

Since λ≤1 the expression in square brackets is negative here, so that $\Phi \left(a^{\prime }\right)<\Phi \left(a\right)$ if ε(>0) is sufficiently small. Hence a does not contain the optimal BLUE coefficients. It follows that the optimal allocation has natural signs when S≤1/(K+1) and so must be a NA.

ii In view of the expression (B2) for the variance of an NA, the optimal allocation for S≤1/(K+1) is the NA, $\pi^{\left(0\right)},$ which minimizes the quantity $F\left(a^{\left(\pi \right)}\right)$, that is
  $$\pi^{\left(0\right)}=\underset{\pi \;\text{is}\;\mathrm{NA}}{\text{argmin}}F\left(a^{\left(\pi \right)}\right).$$

We denote the corresponding BLUE coefficients by $a^{\left(0\right)}=a^{\left(\pi^{\left(0\right)}\right)}=2\pi_{\mathrm{kt}}^{\left(0\right)}\left(2Z_{\mathrm{kt}}-1\right)$. Now let $E\left(S\right)=\mathrm{Eff}\left(\pi^{\left(0\right)}\right)$, considered as a function of S, so that E(S)=1 means that $\pi^{\left(0\right)}$ is an optimum allocation when the ICV =S. Define

$$S_{1}=\sup\left\{S;E\left(S\right)=1\right\}.$$

From (i) above it follows that $S_{1}\geq 1/\left(K+1\right)$ for any layout ℒ and CAC‐matrix, Γ. By definition (of $S_{1}$), we must have E(S)<1 for all $S>S_{1}$. For any $S<S_{1}$ there exists $S^{\prime }>S$, with a corresponding $\lambda^{\prime }>\lambda$, such that $E\left(S^{\prime }\right)=1$. This means that $a^{\left(0\right)}$ minimizes

$$\lambda^{\prime }F\left(a\right)+H\left(a\right)$$

over a∈𝒜. For a∈𝒜, it was shown above (Appendix B(i)) that $H\left(a\right)\geq 4=H\left(a^{\left(\pi \right)}\right)$ for any NA π. Hence

$$\begin{array}{cc} & \lambda F\left(a\right)+H\left(a\right)-\lambda F\left(a^{\left(0\right)}\right)-H\left(a^{\left(0\right)}\right)=\frac{\lambda }{\lambda^{\prime }}\left[\lambda^{\prime }F\left(a\right)+H\left(a\right)-\lambda^{\prime }F\left(a^{\left(0\right)}\right)\right.\\ & \;\left.-H\left(a^{\left(0\right)}\right)\right]+\left(1-\frac{\lambda }{\lambda^{\prime }}\right)\left[H\left(a\right)-H\left(a^{\left(0\right)}\right)\right]\geq 0, \end{array}$$

because both terms are non‐negative. It follows that $a=a^{\left(0\right)}$ minimizes λF(a)+H(a) over a∈𝒜. Hence E(S)=1 for all $S<S_{1}$. Also $E\left(S_{1}\right)=\lim_{S\to S_{1}^{-}}E\left(S\right)=1$, because E(S) is a continuous function of S (see lemma below). Thus $\pi^{\left(0\right)}$ is an optimal allocation if and only if $S\leq S_{1}$.

E(S) *is a continuous function of* S∈(0,1).
We have, by definition,

$$E\left(S\right)=\mathrm{Eff}\left(\pi^{\left(0\right)}\right)=\frac{\min_{a\in 𝒜}\left[\lambda F\left(a\right)+H\left(a\right)\right]}{\lambda F\left(a^{\left(\pi \right)}\right)+4},$$

where λ=KS/(1−S). Clearly the denominator is a continuous function of λ. Define $J\left(\lambda \right)=\min_{a\in 𝒜}\left[\lambda F\left(a\right)+H\left(a\right)\right]$. We will show that J is both left‐ and right‐continuous in λ, for λ>0. It then follows that E(S) is continuous in S. Choose a time epoch t with a Treated cell (k,t) and a Control cell (l,t), and specify a simple allocation, $\pi^{\prime }$, on these cells with BLUE coefficients given by $a_{\mathrm{kt}}^{\prime }=1$ and $a_{\mathrm{lt}}^{\prime }=-1$. For this allocation, $F\left(a^{\prime }\right)=2,$ and $H\left(a^{\prime }\right)=4$. So, for $a={\text{argmin}}_{a\in 𝒜}\left[\lambda F\left(a\right)+H\left(a\right)\right]$ we have $J\left(\lambda \right)=\lambda F\left(a\right)+H\left(a\right)\leq \lambda F\left(a^{\prime }\right)+H\left(a^{\prime }\right)=2\lambda +4$. Since H(a)≥4 and F(a)≥0,this implies that F(a)≤2 and H(a)≤2λ+4. Take a small δ>0. Clearly J(λ) is increasing in λ and

$$\begin{array}{ll} & J\left(\lambda \right)<J\left(\lambda +\delta \right)\leq \min_{𝒜}\left[\left(\lambda +\delta \right)F\left(a\right)+H\left(a\right)\right]=\min_{𝒜}[\lambda F\left(a\right)+H\left(a\right)\\ & \;+\delta F\left(a\right)]\leq J\left(\lambda \right)+2\delta . \end{array}$$

Hence J(λ+δ)→J(λ) as $\delta \to 0^{+}$ so that J(λ) is a right‐continuous function of λ. Similarly, because J(λ)/λ is decreasing in λ we have

$$\frac{J\left(\lambda \right)}{\lambda }<\frac{J\left(\lambda -\delta \right)}{\lambda -\delta }=\min_{𝒜}\left[F\left(a\right)+\frac{H\left(a\right)}{\lambda -\delta }\right]=\min_{𝒜}\left[F\left(a\right)+\frac{H\left(a\right)}{\lambda }+\frac{\delta H\left(a\right)}{\lambda \left(\lambda -\delta \right)}\right]$$

$$\leq \frac{J\left(\lambda \right)}{\lambda }+\frac{\delta }{\lambda \left(\lambda -\delta \right)}\cdot \left(2\left(\lambda -\delta \right)+4\right).$$

Multiplication by (λ−δ) gives

$$\left(1-\frac{\delta }{\lambda }\right)J\left(\lambda \right)<J\left(\lambda -\delta \right)\leq \left(1-\frac{\delta }{\lambda }\right)J\left(\lambda \right)+\delta \left(2+\frac{4-2\delta }{\lambda }\right).$$

Hence J(λ−δ)→J(λ) as $\delta \to 0^{+}$, so that J(λ) is a left continuous function of λ.

## Appendix D Best Natural Allocations for Multiperiod Parallel and Cross‐Over Trials

Suppose there are K=2 cluster sequences over T time‐periods. Because a∈𝒜, we have $a_{2t}=-a_{1t}$ for all t, and $\sum_{t=1}^{T}a_{1t}Z_{1t}+\sum_{t=1}^{T}a_{2t}Z_{2t}=\sum_{t=1}^{T}a_{1t}\left(Z_{1t}-Z_{2t}\right)=1$. Write $a_{1}$ for the vector ${\left(a_{11},a_{12},\ldots ,a_{1T}\right)}^{T}$ and ζ for the vector ${\left(Z_{11}-Z_{12},Z_{21}-Z_{22},\ldots ,Z_{T1}-Z_{T2}\right)}^{T}$. Then the BLUE coefficients for the best NA are obtained by minimizing

$$\sum_{s=1}^{T}\sum_{t=1}^{T}\left(a_{1s}\Gamma_{\mathrm{st}}a_{1t}+a_{2s}\Gamma_{\mathrm{st}}a_{2t}\right)=2a_{1}^{T}\Gamma a_{1}$$

subject to the constraints $\sum_{t=1}^{T}a_{1t}\left(Z_{1t}-Z_{2t}\right)=\zeta^{T}a_{1}=1$, and $\sum_{t=1}^{T}\left(\left|a_{1t}\right|+\left|a_{2t}\right|\right)=2$. If the second constraint is ignored the problem can be solved by a standard Lagrangian method leading to

$$a_{1}={\left(\zeta^{T}\Gamma^{-1}\zeta \right)}^{-1}\Gamma^{-1}\zeta .$$

For a DTD‐model with $\Gamma_{\mathrm{st}}=r^{\left|s-t\right|}$, the inverse of Γ is the tri‐diagonal matrix

$$\Gamma^{-1}={\left(1-r^{2}\right)}^{-1}\left[\begin{array}{lllllll} 1 & -r & 0 & 0 & \cdots & 0 & 0\\ -\;r & 1+r^{2} & -r & 0 & \cdots & 0 & 0\\ 0 & -r & 1+r^{2} & -r & \cdots & 0 & 0\\ \vdots & \vdots & \vdots & \vdots & \ddots & \vdots & \vdots \\ 0 & 0 & 0 & 0 & \cdots & 1+r^{2} & -r\\ 0 & 0 & 0 & 0 & \cdots & -r & 1 \end{array}\right].$$

- If the layout corresponds to a multi‐period parallel study with $Z_{1t}=1$ and $Z_{2t}=0$ for all t, then $\zeta_{t}\equiv 1$ and
  $${\left(\Gamma^{-1}\zeta \right)}^{T}={\left(1-r^{2}\right)}^{-1}\left[1-r,{\left(1-r\right)}^{2},{\left(1-r\right)}^{2},\ldots ,{\left(1-r\right)}^{2},1-r\right]$$

so that

$$a_{1}^{T}\propto \left[1,1-r,1-r,\ldots ,1-r,1\right].$$

Here $\left|\zeta_{t}\right|=1$ and $a_{1t}\zeta_{t}>0$ for all t so that

$$\sum_{k,t}\left|a_{\mathrm{kt}}\right|=2\sum_{t}\left|a_{1t}\right|=2\sum_{t}\left|a_{1t}\right|\left|\zeta_{t}\right|=2\sum_{t}\left|a_{1t}\zeta_{t}\right|=2\left|\sum_{t}a_{1t}\zeta_{t}\right|=2$$

which means that the second constraint is automatically satisfied. Hence the best NA ($\pi_{\mathrm{kt}}\propto \left|a_{\mathrm{kt}}\right|$) is given by

$$\pi_{11}=\pi_{12}=\pi_{T1}=\pi_{T2}=\frac{1}{2\left(2+\left(T-2\right)\left(1-r\right)\right)},$$

$$\pi_{1t}=\pi_{2t}=\frac{1-r}{2\left(2+\left(T-2\right)\left(1-r\right)\right)},\text{for}\;t=2,\ldots ,T-1.$$

b Under a cross‐over layout with $Z_{\mathrm{kt}}=½\left({\left(-1\right)}^{k+t}+1\right)$ we have $\zeta ={\left(-1\right)}^{t+1}$ and a similar argument shows that
  $$a_{1}^{T}\propto \left[1,-\left(1+r\right),1+r,\ldots ,{\left(-1\right)}^{T}\left(1+r\right),{\left(-1\right)}^{T+1}\right]$$

and

$$\pi_{11}=\pi_{12}=\pi_{T1}=\pi_{T2}=\frac{1}{2\left(2+\left(T-2\right)\left(1+r\right)\right)},$$

$$\pi_{1t}=\pi_{2t}=\frac{1+r}{2\left(2+\left(T-2\right)\left(1+r\right)\right)},\text{for}\;t=2,\ldots ,T-1.$$

In both cases (a) and (b), the BLUE coefficients for the best natural allocation automatically minimize the quantity $\sum_{k,t}\left|a_{\mathrm{kt}}\right|$. This means that best natural allocation is optimal for all values of the ICV and $S_{1}=1$.

## Appendix E Optimal Allocation Over the Stepped‐Wedge Layout When $\Gamma =I_{K+1}$

Here, observations from different cluster‐periods are independent of one another and any design consists of a sequence of independent trials at each of K+1 time‐points. However, the first and last time‐points, 0 and K+1, do not entail Treatment/Control comparisons and cannot feature in the optimal design under a model with categorical time‐effects. Each time‐point t=2,…,K contributes a parallel cluster trial with (t−1) Treated cells and (K−t+1) Control cells. The treatment‐effect estimator from such a trial is given by

$$\sigma^{2}\mathrm{var}{\hat{\theta }}_{t}=\rho \left(\frac{1}{t-1}+\frac{1}{K-t+1}\right)+\left(1-\rho \right)\left(\frac{1}{v_{t}}+\frac{1}{w_{t}}\right)$$

where $v_{t}$ is the total number of Treated observations at time t, which, in an optimal layout, are divided equally between the (t−1) Treated cells. Similarly, $w_{t}$ is the number of Control observations which are divided equally between the (K−t+1) Control cells. Then, if $v_{t}+w_{t}=n_{\cdot t}$ is fixed, $\mathrm{var}{\hat{\theta }}_{t}$ is minimized by setting $v_{t}=w_{t}=n_{\cdot t}/2$ and the precision from the trial at time t is given by

$${\left(\mathrm{var}{\hat{\theta }}_{t}\right)}^{-1}=\sigma^{-2}{\left(\frac{K\rho }{\xi_{t}}+\frac{4\left(1-\rho \right)}{n_{\cdot t}}\right)}^{-1},$$

where $\xi_{t}=\left(t-1\right)\left(K-t+1\right)$. Consequently, the precision of the pooled estimator over the (K−1) contributing trials is

$${\left(\mathrm{var}\;\hat{\theta }\right)}^{-1}=\sum_{t=2}^{K}{\left(\mathrm{var}{\hat{\theta }}_{t}\right)}^{-1}=\sigma^{-2}\sum_{t=2}^{K}{\left(\frac{K\rho }{\xi_{t}}+\frac{4\left(1-\rho \right)}{n_{\cdot t}}\right)}^{-1}.$$

This can be maximized over the $n_{\cdot t}$s using the following lemma.
*Suppose that* $x_{i}>0$ and $y_{i}>0$ *for* i=1,…,m. *Then*

$$\sum_{i=1}^{m}{\left(\frac{1}{x_{i}}+\frac{1}{y_{i}}\right)}^{-1}\leq {\left(\frac{1}{\sum_{i}x_{i}}+\frac{1}{\sum_{i}y_{i}}\right)}^{-1}$$

*with equality if and only if* $y_{i}\propto x_{i}$ *for all* i.
Proof of Lemma Define $z_{i}=y_{i}/x_{i}$, and note that z/(1+z) is concave in z for z≥0. Now apply Jensen's inequality over the discrete probability distribution $p_{i}=x_{i}/\sum_{j}x_{j}$. Thus

$$\begin{array}{ll} & E\frac{z_{i}}{1+z_{i}}=\left(\sum_{i}\frac{y_{i}/x_{i}}{1+y_{i}/x_{i}}\cdot x_{i}\right)\cdot {\left(\sum_{i}x_{i}\right)}^{-1}\leq \frac{Ez_{i}}{1+Ez_{i}}\\ & \;=\frac{\left(\sum_{i}\frac{y_{i}}{x_{i}}\cdot x_{i}\right)\cdot {\left(\sum_{i}x_{i}\right)}^{-1}}{1+\left(\sum_{i}\frac{y_{i}}{x_{i}}\cdot x_{i}\right)\cdot {\left(\sum_{i}x_{i}\right)}^{-1}}\cdot \end{array}$$

with equality if and only if $y_{i}/x_{i}$ is constant. Hence

$$\sum_{i}\frac{x_{i}y_{i}}{x_{i}+y_{i}}\leq \frac{\sum_{i}x_{i}\sum_{i}y_{i}}{\sum_{i}x_{i}+\sum_{i}y_{i}},$$

and the result follows.

Using the lemma with $x_{i}=\xi_{i}/K\rho$ and $y_{i}=n_{i\cdot }/\left(4\left(1-\rho \right)\right)$ we find that the precision is maximized when $n_{\cdot t}\propto \xi_{t}=\left(t-1\right)\left(K-t+1\right)$, so that

πkt∝n⋅tt−1∝K−t+1ifk<tn⋅tt−K+1∝t−1ifk≥tfor2≤t≤K,

In agreement with (8). In this case, the pooled estimator has variance

$$\mathrm{var}\;\hat{\theta }=\sigma^{2}\left(\frac{K\rho }{\sum_{t}\xi_{t}}+\frac{4\left(1-\rho \right)}{\sum_{t}n_{t}}\right)=\sigma^{2}\left(\frac{6}{K^{2}-1}\rho +\frac{4}{N}\left(1-\rho \right)\right)$$

as in (9).

## Appendix F The BLUE Coefficients for an Optimal Staircase Design

Here we assume that the optimal solution has staircase support, with notation as in Figure 5. Also define $q_{0}=q_{K}=0$. Then the BLUE coefficients, $q_{1},\ldots ,q_{K-1}$, can be found by minimizing the expression (10) subject to the constraint $\sum_{k=1}^{K-1}q_{k}=1$. Define the Lagrangian $L=\Psi -\delta \sum_{1}^{K-1}q_{k}$, where δ is a Lagrange multiplier. Differentiating L with respect to $q_{k}$ gives

(F1)
$$-\alpha rq_{k+1}+2q_{k}-\alpha rq_{k-1}=\frac{\delta }{2\rho },\text{for}\;k=1,\ldots ,K-1.$$

This difference equation is solved by standard methods.

If αr≠1, the solution may be written in the form

$$q_{k}=Ae^{\left(2k-K\right)\phi }+Be^{\left(K-2k\right)\phi }+C,\text{for}\;k=0,\ldots ,K,$$

where $x=e^{\pm 2\phi }$ are the roots of the auxiliary equation $-\alpha rx^{2}+2x-\alpha r=0$, and A,B,C are constants. On substituting in (F1), it follows that 2(1−αr)C=δ/(2ρ). Note that $\alpha r=\frac{2x}{1+x^{2}}=\mathrm{sech}2\phi$. Applying the boundary conditions $q_{0}=q_{K}=0$ we find that A=B and C=−2AcoshKϕ, from which

(F2)
$${\tilde{q}}_{k}=C\left(1-\frac{\cosh\left(2k-K\right)\phi }{\cosh\mathrm{K\phi}}\right),k=1,\ldots ,K;$$

consistent with (11). The constant C is determined from the constraint $\sum_{k=1}^{K-1}{\tilde{q}}_{k}=1$. Since

$$\sum_{k=1}^{K-1}\cosh\left(2k-K\right)\phi =\frac{\sinh\left(K-1\right)\phi }{\sinh\phi },$$

we have

$$C=\frac{1}{K-\coth\phi \tanh\mathrm{K\phi}}\cdot$$

In (10), the term in ρ is a quadratic form in the $q_{k}$s and is to be minimized subject to a constraint on the sum of the $q_{k}$s. It follows that its minimum value is given by $\left(\delta /2\right)\sum_{k=1}^{K-1}q_{k}=\delta /2$. Hence

$$\sigma^{-2}\mathrm{var}\;\hat{\theta }=\frac{\delta }{2}+\frac{4\left(1-\rho \right)}{N}=\frac{2\left(1-\alpha r\right)\rho }{K-\coth\phi \tanh\mathrm{K\phi}}+\frac{4\left(1-\rho \right)}{N},$$

as in (12). The case αr=1 leads to a pair of repeated roots to the auxiliary equation at x=1. This may be handled directly by trialing a solution of the form $q_{k}=A^{\prime }k^{2}+B^{\prime }k+C^{\prime }$, or by taking a limit as ϕ→0 in (F2) leading to ${\tilde{q}}_{˜k}=6k\left(K-k\right)/K\left(K^{2}-1\right)$, with a corresponding expression for $\mathrm{var}\;\hat{\theta }$. The results are given in the main text, equations (11) and (12).

## Appendix G Conditions for the Staircase Solution to Be Optimal Over a Stepped‐Wedge Layout

Whilst it is relatively straightforward to obtain the optimal BLUE coefficients for a staircase design when the staircase support is assumed, a detailed analysis of perturbations from the solution is needed to find the conditions under which it minimizes the objective function Ψ(.) over the full stepped‐wedge layout. Briefly, we must find the conditions under which a perturbation of the solution that is consistent with the constraints that define 𝒜 will not decrease the objective function Ψ(.)

Because Ψ(.) is a convex function, local optimality is sufficient, and we need consider only infinitesimal perturbations from the solution. However, a difficulty arises because Ψ(a) is not differentiable when a contains zero components, as it does under a staircase support.

We say that the K×T matrix$\;e=\left(e_{\mathrm{kt}}\right)$ is a permissible perturbation (*p.p*.) if $\sum_{k}e_{\mathrm{kt}}=0$ fort=1,…,T and $\sum_{\mathrm{kt}}e_{\mathrm{kt}}Z_{\mathrm{kt}}=0$. Then, for any μ>0, the matrix a+μe is feasible (i.e., satisfies the constraints that define 𝒜) if a itself is feasible and e is a *p.p*. It follows that a feasible matrix a is optimal provided that

$$\underset{\mu \to 0+}{\lim\;\inf}\left[\Psi \left(a+\mu e\right)-\Psi \left(a\right)\right]\geq 0$$

whenever e is a *p.p*.

For any scalar μ>0 we have, from the definition of Ψ(a) in (6), that

Ψ(a+μe)−Ψ(a)=2μΔ(a;e)+o(μ)

where

(G1)
$$\Delta \left(a;e\right)\overset{\mathrm{def}}{=}\rho \;\sum_{k,s,t}a_{\mathrm{ks}}\Gamma_{\mathrm{st}}e_{\mathrm{kt}}+\frac{1-\rho }{N}\left(\sum_{k,t}\left|a_{\mathrm{kt}}\right|\right)\left(\sum_{\left(k,t\right)\in 𝒮}e_{\mathrm{kt}}\mathrm{sign}\left(a_{\mathrm{kt}}\right)+\sum_{\left(k,t\right)\notin 𝒮}\left|e_{\mathrm{kt}}\right|\right),$$

and $𝒮=\left\{\left(k,t\right);a_{\mathrm{kt}}>0\right\}$ is the support of the solution a.

If Δ(a;e)=0, then Ψ(a+μe)−Ψ(a)=o(μ) and it follows from the convexity of Ψ that Ψ(a+μe)≥Ψ(a) for all μ>0. Thus, the condition for optimality is satisfied if and only if Δ(a;e)≥0 for any permissible permutation e.

To investigate the optimality of the staircase solution in (F2) we suppose that $a_{k,k+1}={\tilde{q}}_{k}$ for k=1,…,K−1, and $a_{\mathrm{kk}}=-{\tilde{q}}_{k}$ for k=2,…,K, with $a_{\mathrm{kt}}=0$ elsewhere, so that 𝒮={(t−1,t),(t,t);t=2,…,K}. Throughout the remainder of this section we simplify the notation by suppressing the “~”s and writing $q_{k}$ instead of ${\tilde{q}}_{k}$ for the quantity defined in (F2).

A key point is that any *p.p*., e, can be decomposed into separate *p.p*.s as

(G2)
$$e=f+b+\sum_{t=2}^{K-1}\sum_{k=t+1}^{K}g^{\left(k,t\right)}+c+\sum_{t=3}^{K}\sum_{k=1}^{t-2}h^{\left(k,t\right)}.$$

Here f is a *p*.*p*. on 𝒮 and b is a *p.p*. on the baseline cells {(k,1);k=1,…,K}. The *p.p*. $g^{\left(k,t\right)}$ is defined only when (k,t) lies in the region given by {2≤t<k≤K}. It combines a perturbation in an control cell (k,t) outside the staircase 𝒮 with an equal and opposite perturbation in the nearest cell of the staircase in the vertical direction. Thus $g^{\left(k,t\right)}$ is a *p.p*. on the set {(k,t),(t,t)} defined by $g_{\mathrm{kt}}^{\left(k,t\right)}=e_{\mathrm{kt}}=-g_{\mathrm{tt}}^{\left(k,t\right)}$. Similarly c is a *p.p*. on the endline cells {(k,K+1);k=1,…,K}; and, for 1≤k<t−1<K, $h^{\left(k,t\right)}$ is a *p.p*. on the set {(k,t),(t,t−1)} defined by $h_{\mathrm{kt}}^{\left(k,t\right)}=e_{\mathrm{kt}}=-h_{t,t-1}^{\left(k,t\right)}$. So $h^{\left(k,t\right)}$ combines a perturbation in a treated cell (k,t) outside the staircase 𝒮 with an equal and opposite perturbation in a staircase cell.

The expression in (G1) is linear in e, apart from the $\left|e_{\mathrm{kt}}\right|$ terms, each of which refers to a single cell outside 𝒮. We may write

$$\sum_{\left(k,t\right)\notin 𝒮}\left|e_{\mathrm{kt}}\right|=\sum_{k=1}^{K}\left|e_{k1}\right|+\sum_{t=2}^{K-1}\sum_{k=t+1}^{K}\left|e_{\mathrm{kt}}\right|+\sum_{k=1}^{K}\left|e_{k,K+1}\right|+\sum_{t=3}^{K}\sum_{k=1}^{t-2}\left|e_{\mathrm{kt}}\right|$$

so that it is possible to express Δ(a;e) as a sum over the component *p.p*.s as

$$\begin{array}{ll} & \Delta \left(a;e\right)=\Delta \left(a;f\right)+\Delta \left(a;b\right)+\sum_{t=2}^{K-1}\sum_{k=t+1}^{K}\Delta \left(a;g^{\left(k,t\right)}\right)+\Delta \left(a;c\right)\\ & \;+\sum_{t=3}^{K}\sum_{k=1}^{t-2}\Delta \left(a;h^{\left(k,t\right)}\right). \end{array}$$

It follows that a is a local minimum of Ψ provided that

(G3)
Δ(a;l)≥0

for each sufficiently small *p.p*. of the form $l=f,b,c,g^{\left(k,t\right)}\;\text{or}\;h^{\left(k,t\right)}$.

Note that the property (G3) holds for l=f when a is the staircase solution since a is optimal on 𝒮.

It remains to consider the perturbations $g^{\left(k,t\right)}$ for 2≤t<k≤K, and the permutation, b, on the baseline cells. (Similar results will follow for the $h^{\left(k,t\right)}$s and c, by symmetry, since the problem, and the staircase solution, are invariant under the transformation $k\to K-k;t\to K+2-t;Z_{\mathrm{kt}}\to 1-Z_{\mathrm{kt}}$.

Since Γ is a Toeplitz matrix we can write $\Gamma_{\mathrm{st}}=\Gamma^{\left(\left|s-t\right|\right)}$. Then $\sum_{s=1}^{K+1}a_{\mathrm{ks}}\Gamma_{\mathrm{st}}=\Gamma^{\left(\left|k-t+1\right|\right)}q_{k}-\Gamma^{\left(\left|k-t\right|\right)}q_{k-1}$, where $q_{0}=q_{K}=0$, as usual. Now define

(G4)
$$\Omega \left(t,u\right)=q_{t-1}-\Gamma^{\left(1\right)}q_{t}-\left(\Gamma^{\left(u\right)}q_{t+u-1}-\Gamma^{\left(u+1\right)}q_{t+u}\right)$$

for 1≤t≤K−1,0≤u≤K−t.

Under the EXC‐model (α=r=1), direct calculation with $q_{k}=6k\left(K-k\right)/K\left(K^{2}-1\right)$ shows that $\Omega \left(t,u\right)=-12u/K\left(K^{2}-1\right)$.

First we establish the following general result: Lemma 1
*Necessary and sufficient conditions for the overall optimality of the staircase solution are given by*:

(G5)
Ω(t,u)≤0

(G6)
$$\text{and}\;\Omega \left(t,u\right)\geq -4\frac{1-\rho }{N\rho },$$

*for* 2≤t≤K−1,1≤u≤K−t;

*together with*

(G7)
$$\min_{0\leq k\leq K-1}\Omega \left(1,k\right)-\max_{0\leq k\leq K-1}\Omega \left(1,k\right)\geq -\frac{4\;\left(1-\rho \right)}{N\rho }\cdot$$

*Here the condition* Ω(t,u)≤0 (*for* 2≤t≤K−1,1≤u≤K−t) *is the condition under which the best natural allocation is a staircase, and involves neither the ICC*, ρ, *nor the sample size* N.
Proof of Lemma 1 The conditions (G5) and (G6) are derived by considering the $g^{\left(k,t\right)}$ perturbations, for 2≤t<k≤K. For the staircase solution we have $\sum_{\mathrm{kt}}\left|a_{\mathrm{kt}}\right|=2\sum_{i}q_{i}=2$. Setting $g_{\mathrm{kt}}^{\left(k,t\right)}=\eta =-g_{\mathrm{tt}}^{\left(k,t\right)},$ for some η, and using (G1) we find

$$\Delta \left(a;g^{\left(k,t\right)}\right)=\rho \Omega \left(t,u\right)\eta +\frac{1-\rho }{N}\cdot 2\cdot \left(\eta +\left|\eta \right|\right)$$

with u=k−t. Then the condition for optimality (G3) requires that Ω(t,u)≤0, in case η<0, and $\rho \Omega \left(t,u\right)+4\frac{1-\rho }{N}\geq 0$ in case η>0. This establishes conditions (G5) and (G6). Condition (G7) arises on considering a *p.p*. on the baseline cells—that is $b_{k1}=\eta_{k}$ for k=1,…,K with $\sum \eta_{k}=0$. We have

$$\Delta \left(a;b\right)=\rho \sum_{k=1}^{K}\left(\Gamma^{\left(k\right)}q_{k}-\Gamma^{\left(k-1\right)}q_{k-1}\right)\eta_{k}+2\frac{1-\rho }{N}\sum_{k=1}^{K}\left|\eta_{k}\right|.$$

Now let $H=\sum_{\eta_{k}>0}\eta_{k}$ so that $\sum_{k}\left|\eta_{k}\right|=2H$ and H>0. On considering the least favorable distribution for the values of $\eta_{k}$, we find

(G8)
$$\Delta \left(a;b\right)\geq \rho \left[\min_{k}\left(\Gamma^{\left(k\right)}q_{k}-\Gamma^{\left(k-1\right)}q_{k-1}\right)-\max_{k}\left(\Gamma^{\left(k\right)}q_{k}-\Gamma^{\left(k-1\right)}q_{k-1}\right)\right]H+4\frac{1-\rho }{N}H,$$

with equality for a *p.p*. confined to the cells corresponding to the minimum and maximum values of$\;\Gamma^{\left(k\right)}q_{k}-\Gamma^{\left(k-1\right)}q_{k-1}$. The condition for optimality is satisfied if and only if the right‐hand side of (G8) is non‐negative. The required result (G7) follows from the definition of Ω(1,k) in (G4) on dividing (G8) by H.■

### Explicit Conditions for the Γ‐Family

Lemma 1 gives conditions for a staircase to be optimal under a general Toeplitz CAC‐matrix.

Hereafter we suppose that Γ takes the particular form in Equation (1) of the main paper namely

$$\Gamma_{\mathrm{st}}=\Gamma^{\left(\left|s-t\right|\right)}=\left\{\begin{array}{ll} 1, & s=t\\ \alpha r^{\left|s-t\right|}, & s\neq t \end{array}\right.,$$

for some (α,r)∈[0,1]×[0,1], though excluding the case α=r=1 which is postponed to a later section.

We establish two preliminary results for CAC‐matrices of this form. Lemma 2a
*If* Γ *takes the form described in* (1), *then*

$$\min_{1\leq t\leq K-1}\Omega \left(t,K-t\right)=\Omega \left(1,K-1\right).$$

When α=r=1, the result follows at once because $\Omega \left(t,u\right)=-3u/K\left(K^{2}-1\right)$. Otherwise, from the definition of Ω, and noting that $q_{K-1}=q_{1}$ and $q_{K}=q_{0}=0$, we have

$$\Omega \left(t,K-t\right)=q_{t-1}-\alpha rq_{t}-\left({\alpha r}^{K-t}q_{K-1}-{\alpha r}^{K-t+1}q_{K}\right)=q_{t-1}-\alpha rq_{t}-{\alpha r}^{K-t}q_{1}.$$

Now set $\omega_{t}=q_{t}-\alpha rq_{t-1}$. The required result will follow if

(G9)
$$\omega_{t}\geq \alpha q_{1}\left(r^{K-t}-r-r^{K-1}\right),\text{for}\;1\leq t\leq K-1.$$

From (F2), or (11), we have

$$\omega_{t}\propto \cosh\mathrm{K\phi}-\cosh\left(2t-2-K\right)\phi -\mathrm{sech}\left(2\phi \right)\left[\cosh\mathrm{K\phi}-\cosh\left(2t-K\right)\phi \right].$$

On applying the identity

cosh(2t−K)ϕ=cosh(2t−K)ϕcosh2ϕ+sinh(2t−2−K)ϕsinh2ϕ

$\text{with}\;\cosh2\phi =1+2\sinh^{2}\phi \;\text{andsinh}2\phi =2\sinh\phi \cosh\phi ,$
We find that

$$\omega_{t}\propto 2\sinh\phi \mathrm{sech}2\phi \left[\cosh\mathrm{K\phi}\sinh\phi +\sinh\left(2t-2-K\right)\phi \cosh\phi \right].$$

Since ϕ≥0, it follows that $\omega_{t}\geq 0$ when t≥1+K/2. Moreover, the right‐hand side of (G9) is <0 for all t≤K−1 so that the inequality (G9) holds whenever 1+K/2≤t≤K−1. For 1≤t≤1+K/2 we define the (continuous) function $h\left(t\right)=\alpha q_{1}\left(r^{K-t}-r-r^{K-1}\right)-\omega_{t}$. It follows that h(1)=0, h(1+K/2)≤0 and a simple calculation shows that h(t) has an increasing first derivative over the range 1≤t≤1+K/2. Hence h(t) is a convex function of t on this range and

$$h\left(t\right)\leq \frac{t}{K/2}\cdot h\left(1\right)+\frac{K/2-t}{K/2}\cdot h\left(1+\frac{K}{2}\right)\leq 0,$$

as required.
Lemma 2b
*Suppose that* α *and* r *are chosen such that* Ω(t,1)≤0 *for all* 2≤t≤K−1. *Then* Ω(t,u) *is a decreasing function of* u *for* 1≤t≤K−1, *with* 0≤u≤K−t.
Using the expression for $q_{k}$ in (F2) we have $q_{2}<2q_{1}\leq \frac{2}{r}q_{1}$. Since $q_{0}=0$, it follows that $\Omega \left(1,1\right)=-2\alpha rq_{1}+\alpha r^{2}q_{2}<0$ so that the hypothesis Ω(t,1)≤0 applies when 1≤t≤K−1. From the definition of Ω, we have

$$\Omega \left(t,u+1\right)-\Omega \left(t,u\right)=\Gamma^{\left(u\right)}q_{t+u-1}-\Gamma^{\left(u+1\right)}q_{t+u}-\left(\Gamma^{\left(u+1\right)}q_{t+u}-\Gamma^{\left(u+2\right)}q_{t+u+1}\right)$$

$$\leq r^{u}q_{t+u-1}-\alpha r^{u+1}q_{t+u}-\left(\alpha r^{u+1}q_{t+u}-\alpha r^{u+2}q_{t+u+1}\right)=r^{u}\Omega \left(t+u,1\right)\leq 0,$$

by hypothesis. Hence Ω(t,u) is decreasing in u as claimed.

It follows immediately from Lemma 2b that $\min_{0\leq u\leq K-t}\Omega \left(t,u\right)=\Omega \left(t,K-t\right)$ and $\max_{0\leq u\leq K-t}\Omega \left(t,u\right)=\Omega \left(t,0\right)=0$ whenever Ω(t,1)≤0 for all 2≤t≤K−1.

Lemmas 2a and 2b imply that the optimality conditions in Lemma 1 can be expressed in the following form: Lemma 3
*If* $\Gamma^{\left(s\right)}=\alpha r^{s}$, *for* s≥1, *the following conditions are necessary and sufficient for the overall optimality of the staircase solution*:

(G10)
$$\max_{2\leq t\leq K-1}\Omega \left(t,1\right)\leq 0$$

*and*

(G11)
$$\Omega \left(1,K-1\right)\geq -4\frac{1-\rho }{N\rho }\equiv -\frac{4}{K}\cdot \frac{1-S}{S}.$$

Here (G10) is the best‐natural condition, which is (necessarily) independent of N and ρ. It is clearly satisfied under the EXC‐model since $\Omega \left(t,1\right)=-12/K\left(K^{2}-1\right)$ for all t in this case. Hence the best natural allocation is a staircase under the EXC‐model.

The second condition (G11) implies that $S_{1}=4/\left(4-K\Omega \left(K-1,1\right)\right)$. Proof of Lemma 3 We show that conditions (G10) and (G11) together are equivalent to conditions (G5)–(G7) in Lemma 1. If αr=1, Ω(t,u)∝−u, and the result follows immediately by direct calculation. Now consider the case αr<1. First assume condition (G10) and apply Lemma 2b. It follows that$\max_{0\leq k\leq K-1}\Omega \left(1,k\right)=\Omega \left(1,0\right)=0$ and $\min_{0\leq k\leq K-1}\Omega \left(1,k\right)=\Omega \left(1,K-1\right)$ so that condition (G7) is equivalent to condition (G11); furthermore,
$\min_{t\geq 2}\;\min_{u\leq K-t}\Omega \left(t,u\right)=\min_{t\geq 2}\Omega \left(t,K-t\right)\geq \min_{t\geq 1}\Omega \left(t,K-t\right)=\Omega \left(1,K-1\right)$, from Lemma 2a. Thus condition (G6) is redundant since it is implied by condition (G7). Hence (G10) and (G11) together imply (G5)–(G7). On the other hand, it is clear that condition (G5) implies (G10) and the argument of the previous paragraph may be used to show that (G5)–(G7) together imply (G10) and (G11). ■

To obtain explicit conditions for optimality in terms of the CAC‐parameters α and r, we apply Lemma 3 with $\Omega \left(t,1\right)=q_{t}-2\alpha rq_{t+1}+\alpha r^{2}q_{t+2}$. Now substitute $\alpha rq_{t+2}=2q_{t+1}-\alpha rq_{t}-\frac{\delta }{2\tau^{2}}$, from the difference Equation (F1) for $q_{t}$, to get

$$\Omega \left(t,1\right)=\left(1-\alpha r^{2}\right)q_{t}+2r\left(1-\alpha \right)q_{t+1}+\text{constant}.$$

Here the coefficients $\left(1-\alpha r^{2}\right)$ and (1−α) are both >0. Thus Ω(t,1) is proportional to a constant term plus a convex combination of $q_{t}$ and $q_{t+1}$. When $q_{t}$ is considered as a continuous function of t, $\left[\text{that is}\;q_{t}\propto \cosh\mathrm{K\phi}-\cosh\left(2t-K\right)\phi \right]$ it is concave and symmetrical about its maximum at $t=\frac{K}{2}$. It follows that, when K is odd, the greatest value of Ω(t,1) occurs when t and t+1 straddle $\frac{K}{2}$, that is at the point $t_{0}=\frac{K-1}{2}$. If K is even there are two cases to consider, depending on which of the coefficients in the convex combination is greatest. In summary,

If K is odd,

$$t_{0}=\underset{t}{\text{argmax}}\Omega \left(t,1\right)=\frac{\left(K-1\right)}{2};$$

and if K is even,

$$t_{0}=\underset{t}{\text{argmax}}\;\Omega \left(t,1\right)=\left\{\begin{array}{ll} \frac{K}{2}, & \text{if}\;1-\alpha r^{2}\geq 2\left(1-\alpha \right)r\\ \frac{K}{2}-1, & \text{if}\;1-\alpha r^{2}\leq 2\left(1-\alpha \right)r \end{array}\right.$$

The best natural conditions in the main text (13) and (14) are obtained, after some manipulation, from the inequality $\Omega \left(t_{0},1\right)\leq 0$, using the formula

$$\begin{array}{cc} & \Omega \left(t,1\right)\propto \left(1-2\alpha r+\alpha r^{2}\right)\cosh\mathrm{K\phi}-\cosh\left(2t-K\right)\phi \\ & \;+2\alpha r\;\cosh\left(2t+2-K\right)\phi -\alpha r^{2}\cosh\left(2t+4-K\right)\phi . \end{array}$$

The second condition, for full optimality (15), follows directly from (G11) above, using the definition of Ω in (G4), which gives the formula

$$\Omega \left(K-1,1\right)=q_{0}-\alpha rq_{1}-\left(\alpha r^{K-1}q_{K-1}-\alpha r^{K}q_{K}\right)=-\alpha rq_{1}\left(1+r^{K-2}\right)$$

since $q_{0}=q_{K}=0$ and $q_{1}=q_{K-1}$.

## Appendix H Optimal Allocations Over the Stepped‐Wedge Layout Under the Exchangeable Model

Under the EXC model (α,r)=(1,1)) a complete solution to the optimal design problem for a stepped‐wedge layout is available for all values of the ICV.

From Appendix F, the best natural allocation has staircase support with

$$\pi_{k,k+1}^{\left(0\right)}=\pi_{k+1,k+1}^{\left(0\right)}=\frac{3k\left(K-k\right)}{K\left(K^{2}-1\right)},k=1,\ldots ,K-1,$$

and is optimal when $S\leq S_{1}=\left(K+1\right)/\left(K+4\right)$.

For S>(K+1)/(K+4), we show that:

Any optimal allocation, $\tilde{\pi }$, can be expressed as a convex combination of the best natural (staircase) allocation, $\pi^{\left(0\right)}$, with an allocation $\pi^{\left(1\right)}$ described below. We have

$${\tilde{\pi }}_{\mathrm{kt}}=\psi \pi_{\mathrm{kt}}^{\left(0\right)}+\left(1-\psi \right)\pi_{\mathrm{kt}}^{\left(1\right)},k=1,\ldots K,t=1,\ldots ,K+1,$$

where ψ=⅓(K+1)(1−S)/S and $\pi^{\left(1\right)}$ satisfies

$$\pi_{k,k+1}^{\left(1\right)}=\pi_{k+1,k+1}^{\left(1\right)}=\frac{1}{2K},k=1,\ldots ,K-1;$$

$$\pi_{11}^{\left(1\right)}+\pi_{1,K+1}^{\left(1\right)}=\frac{1}{2K},\pi_{K1}^{\left(1\right)}=\pi_{11}^{\left(1\right)},\pi_{K,K+1}^{\left(1\right)}=\pi_{1,K+1}^{\left(1\right)},$$

$$\pi_{\mathrm{kt}}^{\left(1\right)}=0\;\text{in}\;\mathrm{all}\;\text{other cells}.$$

The efficiency of $\pi^{\left(0\right)}$ is

$$\mathrm{Eff}\left(\pi^{\left(0\right)}\right)={\left[1+\frac{K-1}{K^{2}}\left(\psi +\psi^{-1}-2\right)\right]}^{-1}.$$

An example is given in Figure 7, which shows the best natural allocation for 6 clusters, together with the skew‐symmetric version of $\pi^{\left(1\right)}$, and a fully optimal allocation when $S=\frac{7}{8}$, which is greater than the threshold $S_{1}=0.7$ in this case. Here the optimal allocation entails ‘hotspots’ in the corner of the layout, in addition to the staircase cells. As S→1 the optimal allocation approaches $\pi^{\left(1\right)}$. This would arise for fixed N when ρ→1, so that $\pi^{\left(1\right)}$ is actually an optimal allocation under a model with fixed cluster effects. As expected, the efficiency of $\pi^{\left(0\right)}$ decreases to 0 as S tends to 1—though this effect is less immediate for larger values of K.

1. To begin with we assume that the optimal allocation is a staircase augmented by the addition of the corner cells {(1,1),(1,K+1),(K,1),(K,K+1)} with $a_{K1}=-a_{11}=\nu_{0}/2$ and $a_{K,K+1}=-a_{1,K+1}=\nu_{K}/2$ and seek a local optimum of the objective function (6) for which $\nu_{0}>0$, $\nu_{K}>0$ and $q_{k}>0$ for k=1,…,K−1. Now set $q_{K}=q_{0}=\left(\nu_{0}+\nu_{K}\right)/2$. The objective function takes the form

(H1)
$$\Psi \left(a\right)=\rho \sum_{k=0}^{K-1}\left(q_{k}^{2}-2q_{k}q_{k+1}+q_{k+1}^{2}\right)+\frac{4\left(1-\rho \right)}{N}{\left(1+q_{0}\right)}^{2},$$

so that the value of Ψ(a) depends on the corner cells only through the average value of the ν‐coefficients. This feature leads to non‐uniqueness of the solution. For the skew‐symmetric solution we put $\nu_{0}=\nu_{K}=q_{0}/2$. The constraint $\sum a_{\mathrm{kt}}Z_{\mathrm{kt}}=1$ means that $\sum_{1}^{K-1}q_{k}=1$, so we minimize the Lagrangian $L=\Psi -\delta \sum_{1}^{K-1}q_{k}$. Differentiating L with respect to $q_{k}$ for k=1,…,K−1 leads to

(H2)
$$-{\tilde{q}}_{k+1}+2{\tilde{q}}_{k}-{\tilde{q}}_{k-1}=\frac{\delta }{2\rho },\text{for}\;k=1,\ldots ,K-1.$$

If we take ${\tilde{q}}_{0}={\tilde{q}}_{K}=0$ with $\sum_{1}^{K-1}{\tilde{q}}_{k}=1$, then the difference equation is satisfied by the staircase solution with

$$q_{k}^{\left(0\right)}=\frac{6k\left(K-k\right)}{K\left(K^{2}-1\right)},k=0,1,\ldots ,K.$$

In the present case ${\tilde{q}}_{0},{\tilde{q}}_{K}$ are non‐zero but the constraint $\sum_{1}^{K-1}{\tilde{q}}_{k}=1$ still holds. Then the solution to (H2) can be written in the form

$${\tilde{q}}_{k}=\zeta q_{k}^{\left(0\right)}+\left(1-\zeta \right)\frac{1}{K-1},k=0,\ldots ,K$$

for some constant ζ. Setting k=0 and k=1 gives

(H3)
$${\tilde{q}}_{0}={\tilde{q}}_{K}=\left(1-\zeta \right)\frac{1}{K-1}\;\text{and}\;{\tilde{q}}_{1}={\tilde{q}}_{K-1}=\zeta q_{1}^{\left(0\right)}+\left(1-\zeta \right)\frac{1}{K-1}$$

since $q_{0}^{\left(0\right)}=q_{K}^{\left(0\right)}=0$ and $q_{1}^{\left(0\right)}=q_{K-1}^{\left(0\right)}$. Differentiating L w.r.t. $q_{0}$ we find

(H4)
$$2\rho \left(-{\tilde{q}}_{1}+2{\tilde{q}}_{0}-{\tilde{q}}_{K-1}\right)=4\rho \left({\tilde{q}}_{0}-{\tilde{q}}_{1}\right)=-\frac{8\left(1-\rho \right)}{N}\left(1+{\tilde{q}}_{0}\right).$$

Combining (H3) with (H4) leads to

$$\frac{N\rho }{2\left(1-\rho \right)}q_{1}^{\left(0\right)}\zeta -\left(1-\zeta \right)\frac{1}{K-1}=1$$

from which

$$\zeta =\frac{K}{1+\psi^{-1}\left(K-1\right)},$$

where ψ=⅓(K+1)(1−S)/S. Since $S>S_{1}$, it follows that ψ<1 and 0≤ζ<1. Thus, the solution obtained is consistent with the initial assumption that $q_{k}>0$, for k=0,…,K, and must correspond to a global optimum. To obtain the optimal allocation using (5), we first compute

$$\sum_{k,t}\left|a_{\mathrm{kt}}\right|=2\sum_{k=0}^{K-1}q_{k}=2\left[\zeta \sum_{k=1}^{K-1}q_{k}^{\left(0\right)}+\left(1-\zeta \right)\cdot \frac{K}{K-1}\right]=2\cdot \frac{K-\zeta }{K-1}.$$

But ψ=(K−1)ζ/(K−ζ), so that

$$\begin{array}{cc} & {\tilde{\pi }}_{k,k+1}={\tilde{\pi }}_{˜k+1,k+1}=\frac{q_{k}}{\sum \left|a_{\mathrm{lt}}\right|}=\frac{K-1}{2\left(K-\zeta \right)}\left(\zeta q_{k}^{\left(0\right)}+\left(1-\zeta \right)\cdot \frac{1}{K-1}\right)\\ & \;=\psi \pi_{k,k+1}^{\left(0\right)}+\left(1-\psi \right)\cdot \frac{1}{2K}=\psi \pi_{k,k+1}^{\left(0\right)}+\left(1-\psi \right)\pi_{k,k+1}^{\left(1\right)} \end{array}$$

as claimed. ■

2 The variance of the BLUE of θ under the augmented staircase solution can be found from the Lagrange multiplier δ, on noting that the expression for Ψ(.) in (H1) is a quadratic form in the variables $\left\{\left(1+q_{i}\right);i=1,\ldots ,K-1\right\}$, to be minimized subject to the constraint $\sum_{1}^{K-1}\left(1+q_{i}\right)=K$. Here $\delta {\left(2\rho \right)}^{-1}=-q_{k+1}+2q_{k}-q_{k-1}=12\zeta {\left(K\left(K^{2}-1\right)\right)}^{-1}$. After a little algebra, this gives
  $$\begin{array}{cc} & \mathrm{var}\;\hat{\theta }=\sigma^{2}\Psi \left(\tilde{a}\right)=\frac{\delta }{2}K\sigma^{2}=\rho \zeta \frac{12}{K\left(K^{2}-1\right)}K\sigma^{2}=\frac{4\left(1-\rho \right)}{N}{\left(\frac{K}{K-1}\right)}^{2}\\ & \;{\left(1+\frac{\phi }{K-1}\right)}^{-1}\sigma^{2}. \end{array}$$

The formula for $\mathrm{Eff}\left(\pi^{\left(0\right)}\right)$ follows on comparison with the variance expression (12) in the main paper.■ It remains to verify that the allocation (20) is actually optimal over the full stepped‐wedge layout by introducing permissible perturbations, e, of the BLUE coefficients, a, for the augmented staircase solution. A similar decomposition to that in equation (G2) applies with f confined to the augmented staircase support, and the baseline perturbation given by

$$b=\sum_{k=2}^{K-1}b_{k}$$

where $b_{k}$ consists of a perturbation η in row k (2≤k≤K−1)balanced by a perturbation −η in row 1. (A similar decomposition applies to the endline term c.) As before, we need only consider perturbations that involve unoccupied cells of the layout—that is outside the augmented staircase—because the solution is optimized over the augmented staircase support. Consider first the baseline perturbation $b_{k}$. (Effectively a $g^{\left(k,t\right)}$ perturbation with t=1.) The corresponding first order change in Ψ is

$$\Delta \left(a;e\right)=\left\{\begin{array}{ll} 2\rho \left(q_{0}-q_{1}+q_{k}-q_{k-1}\right)\eta , & \text{if}\;\eta <0\\ 2\rho \left(q_{0}-q_{1}+q_{k}-q_{k-1}\right)\eta +\frac{8\left(1-\rho \right)}{N}\left(1+q_{0}\right)\eta , & \text{if}\;\eta >0 \end{array}\right.$$

But $q_{0}-q_{1}+q_{k}-q_{k-1}=12\zeta \left(1-k\right)/\left(K\left(K^{2}-1\right)\right)<0$, so that Δ(a;e)>0 if η<0. For η>0 we have

$$\Delta \left(a;e\right)>2\rho \left(q_{0}-q_{1}+q_{K}-q_{K-1}\right)\eta +\frac{8\left(1-\rho \right)}{N}\left(1+q_{0}\right)\eta$$

$$=4\rho \left(q_{0}-q_{1}\right)\eta +\frac{8\left(1-\rho \right)}{N}\left(1+q_{0}\right)\eta =0,$$

on setting $q_{K-1}=q_{1}$ and using (H4). Similarly, the effect of a $g^{\left(k,t\right)}$ perturbation with k>t is given by

$$\Delta \left(a;e\right)=\left\{\begin{array}{ll} 2\rho \left(q_{t-1}-q_{t}+q_{k}-q_{k-1}\right)\eta =4\rho \left(t-k\right)\eta >0, & \text{if}\;\eta <0\\ 2\rho \left(q_{t-1}-q_{t}+q_{k}-q_{k-1}\right)\eta +\frac{8\left(1-\rho \right)}{N}\left(1+q_{0}\right)\eta , & \text{if}\;\eta >0 \end{array}\right.$$

Here $\left(q_{t-1}-q_{t}+q_{k}-q_{k-1}\right)>q_{0}-q_{1}+q_{K}-q_{K-1}=2\left(q_{0}-q_{1}\right)$, so that Δ(a;e)>0 when η>0, as before. Hence, any small perturbation of the augmented staircase solution leads to an increase in the objective function, and the optimality of the solution is established.

## Appendix I The BLUE Coefficients for Step 1 of the Algorithm

For any specified design $\left(n_{\mathrm{kt}}\right)$ the BLUE of θ is equivalent to the generalized least squares estimator (GLSE). Here we take $n_{\mathrm{kt}}$ to be defined on the complete K×T lattice, with $n_{\mathrm{kt}}=0$ for all (k,t)∉ℒ.

Define the K×Tmatrix Y of cell‐means by

$$Y_{\mathrm{kt}}=\left\{\begin{array}{l} \frac{1}{n_{\mathrm{kt}}}\sum_{i}y_{\mathrm{kti}},\text{if}\;n_{\mathrm{kt}}\neq 0\\ 0,\text{if}\;n_{\mathrm{kt}}=0 \end{array}\right..$$

The model for Y may be written as:

$$E\mathrm{vec}\left(Y^{T}\right)=X\omega$$

with (block‐diagonal) variance–covariance matrix

$$V=\tau^{2}\left(I_{K}⊗\Gamma \right)+\sigma^{2}D^{-1}.$$

Here $X=\left[\mathrm{vec}\left(Z^{T}\right)\vdots 1_{K}⊗I_{T}\right]$ is aKT×(T+1) patterned matrix, $\omega =\left[\begin{array}{l} \theta \\ \beta \end{array}\right]$ is a (T+1)×1 vector of parameters and $D=\mathrm{diag}\left(\mathrm{vec}\left(n^{T}\right)\right)$. The elements of $D^{-1}$ corresponding to empty cells in the layout are notionally infinite and the corresponding rows and columns of V are undefined. However, $\mathrm{DV}=\tau^{2}D\left(I_{K}⊗\Gamma \right)+\sigma^{2}I_{\mathrm{KT}}$ is a well‐defined matrix, and the estimate of ω can be written as

$$\hat{\omega }={\left(X^{T}{\left(\mathrm{DV}\right)}^{-1}\mathrm{DX}\right)}^{-1}X^{T}{\left(\mathrm{DV}\right)}^{-1}D\;\mathrm{vec}\left(Y^{T}\right).$$

The coefficients for $\hat{\theta }$ – that is ${\left(\mathrm{vec}a^{T}\right)}^{T}$—are contained in the first row of the (T+1)×KT matrix

$${\left(X^{T}{\left(\mathrm{DV}\right)}^{-1}\mathrm{DX}\right)}^{-1}X^{T}{\left(\mathrm{DV}\right)}^{-1}D.$$
